# Short-term variability of the human serum metabolome depending on nutritional and metabolic health status

**DOI:** 10.1038/s41598-020-72914-7

**Published:** 2020-10-01

**Authors:** Inoncent Agueusop, Petra B. Musholt, Beate Klaus, Kendra Hightower, Aimo Kannt

**Affiliations:** 1 Sanofi Research and Development, Frankfurt, Germany; 2 Nuvisan GmbH, Neu-Ulm, Germany; 3 Metabolon Inc., Morrisville, USA; 4 Experimental Pharmacology, Medical Faculty Mannheim, University of Heidelberg, Mannheim, Germany; 5 Fraunhofer IME-TMP, Frankfurt, Germany

**Keywords:** Metabolism, Diabetes, Obesity, Biomarkers

## Abstract

The intra-individual variability of the human serum metabolome over a period of 4 weeks and its dependence on metabolic health and nutritional status was investigated in a single-center study under tightly controlled conditions in healthy controls, pre-diabetic individuals and patients with type-2 diabetes mellitus (T2DM, n = 10 each). Untargeted metabolomics in serum samples taken at three different days after overnight fasts and following intake of a standardized mixed meal showed that the human serum metabolome is remarkably stable: The median intra-class correlation coefficient (ICC) across all metabolites and all study participants was determined as 0.65. ICCs were similar for the three different health groups, before and after meal intake, and for different metabolic pathways. Only 147 out of 1438 metabolites (10%) had an ICC below 0.4 indicating poor stability over time. In addition, we confirmed previously identified metabolic signatures differentiating healthy, pre-diabetic and diabetic individuals. To our knowledge, this is the most comprehensive study investigating the temporal variability of the human serum metabolome under such tightly controlled conditions.

## Introduction

Circulating metabolites are assessed because they may be indicative of the body’s responses to nutrition, disease, treatment or environmental factors. Metabolites have potential to be used e.g. for predicting disease progression, response to therapy, demonstrating drug target engagement in clinical trials or for prognostic purposes. There is an increasing body of evidence for using metabolomics in medicine, such that the field is now poised to discover clinically useful biomarkers and therapeutic targets in nephrology, cancer, and other medical fields^1,2^. With this growing interest to perform metabolomics analyses for biomarker identification, it is crucial to accurately understand the diurnal variation, the course over time per subject, as well as the intra- and inter-subject variability also dependent on metabolic condition or nutritional status among other factors^3^. The biological interpretation of biomarkers with large fluctuation will be different from the interpretation of biomarkers which are perceived or known to be stable.

Published studies on metabolites are frequently completed on cohorts with a small number of subjects included, a limited number of biological samples collected per subject and over time, and are based on targeted approaches with a limited number of metabolites investigated. In addition, samples are often collected at different clinical centers and by different investigators, and potentially in subjects with unclear nutritional status. This may lead to biased estimates of the intra- but also inter-subject variability and has the inherent weakness that markers showing high variability are more likely to be identified as “significantly changing” just by chance, in comparison to markers which are stable over time. Furthermore, the populations in which biomarkers are investigated are often not homogenous or not sufficiently characterized. Kim et al.^4^ performed non-targeted metabolomics analysis of blood and urine of healthy subjects over a short period of time and suggested that blood and urine are suitable biofluids for metabolomics studies. They investigated the source of variability attributed to technical issues such as sample preparation and analysis. Most of the available studies on diet and metabolomics have focused on the effects of a specific dietary intervention^5,6^ rather than day-to-day variability.

The identification and validation of known or new biomarkers—that, for example, predict disease progression or response to treatment, demonstrate target engagement or are indicative of disease type, severity or a specific mode of action—is a key success factor to assess the clinical relevance of a particular treatment approach. Biomarkers which are subject to high variability, either if assessed over a defined observation period between individuals, or within individuals if repeatedly measured, may have only limited use in clinical practice. Especially the evaluation of effects of disease-modifying treatments through the application of a biomarker or a panel of biomarkers strongly requires knowledge about the characteristics of these markers under conditions where no treatment intervention was applied. Characterization and validation of a biomarker could be best achieved if multiple samples over a defined sampling period from one subject are tested, as well as a comparison of samples from different subjects. If the purpose is also to test the ability of a biomarker to predict disease modification or progression, then samples from different populations (with/without disease) should be collected in addition.

We have designed and performed a study aiming at investigating the intra-individual variability of the human serum metabolome over a period of 28 days, while eliminating as many of the potential sources of variability as possible: The study was performed in a single-center setting with repeated blood sample collection on three different days under carefully controlled conditions. Participants stayed overnight before each study day to control food intake. Serum samples from 30 min before and 1 h after intake of a standardized mixed meal were used to perform untargeted global metabolomics and lipidomics analyses to (a) investigate the intra-individual variability of serum metabolites over time, (b) its dependence on nutritional status and metabolic health, and (c) identify metabolic signatures of healthy, prediabetic and T2DM individuals and potential differences in response to a mixed meal. To our knowledge, this is the first and most comprehensive study investigating the variability of the human serum metabolome under tightly controlled conditions over a short period of time in subjects with different metabolic health conditions and controlled nutritional status. We collected over 40.000 samples in different matrices (serum, EDTA-, Li-heparin-, NaF- and p800-plasma) and different body fluids including urine. These samples are available for further biomarker analyses.

## Results

### Study participants

**Figure 1:**
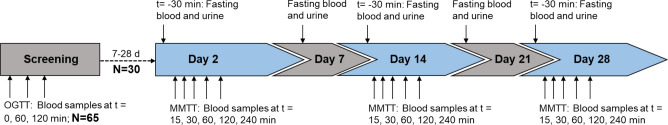
Graphical study design. Grey: ambulatory visits. Blue: in-house study days with participants staying the night before the study day at the study center.

**Figure 2:**
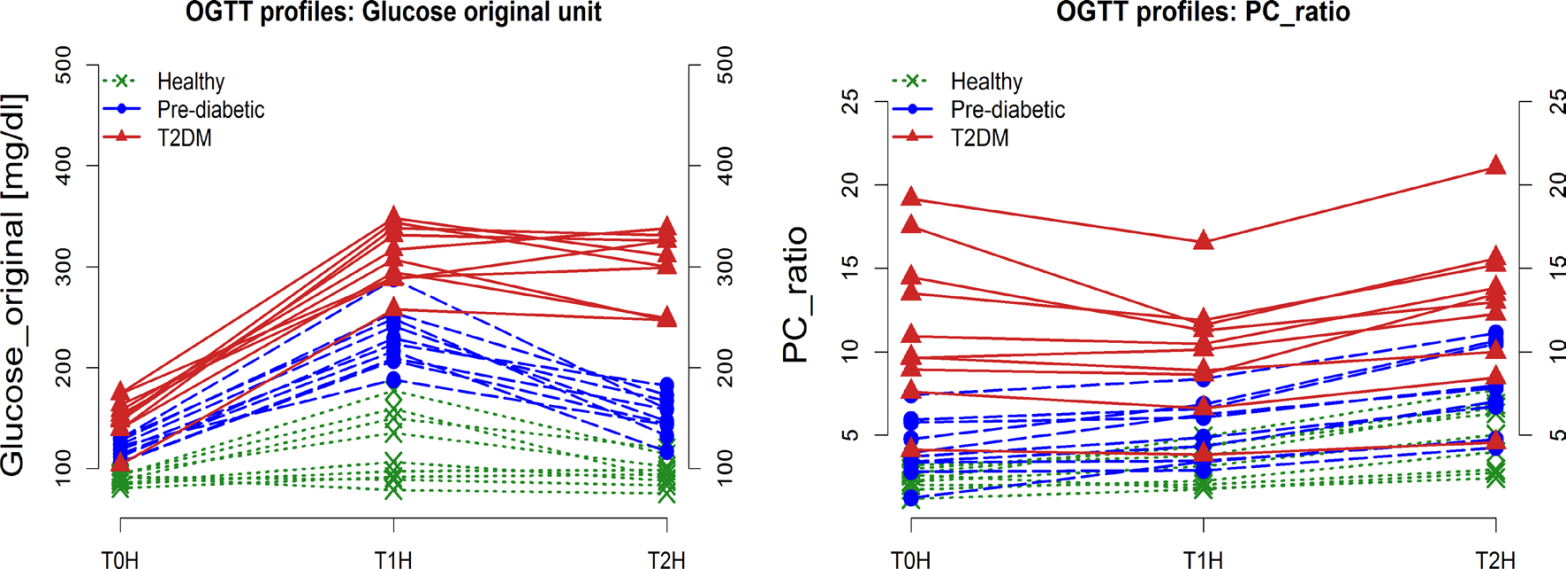
OGTT glucose (*left*) and proinsulin:c-peptide (PC) ratio profile (*right*) for subjects included into the study (N = 30). Graphs are colored by health group.

The overall study layout is depicted in Fig. 1. Three groups of individuals (n = 10 each) were recruited for the study: healthy subjects, individuals with pre-diabetes and patients with T2DM. Assignment to the three groups was done based on fasting glucose, and glycated hemoglobin (HbA1c) according to ADA criteria^7^ as well as the results of the OGTT-challenged 1-h and 2-h glucose, intact proinsulin and C-peptide, and the intact proinsulin/C-peptide (PC) ratio. The values for the different parameters are provided in Table 1 by subgroup. Plasma glucose and proinsulin: c-peptide (PC) ratios are depicted in Fig. 2. As reflected by use of ADA criteria for group allocation, the OGTT glucose profiles clearly distinguish the three study groups.

**Table 1:** Fasting screening data for the study population (N = 30).

	Healthy individuals (N = 10)	Pre-diabetic sindividuals (N = 10)	T2DM patients (N = 10)
HbA1c (%)	5.40^b^ (0.31^a^)	5.86 (0.34)	7.46 (0.59)
FPG (mmol/L)	4.95 (0.27)	6.61 (0.51)	8.33 (1.14)
C-Peptide (nmol/L)	0.58 (0.2)	1.53 (0.94)	1.28 (0.44)
Proinsulin (pmol/L)	1.25 (0.0)	6.4 (4.23)	14.64 (6.75)
PC-ratio	2.34 (0.6)	4.24 (1.77)	11.55 (4.6)

*FPG* fasting plasma glucose, *PC-ratio* proinsulin/C-peptide ratio.

^a^Standard deviation.

^b^Mean.

The interpretation of the wide range of PC ratios for T2DM patients after an OGTT challenge, on the other hand, needs to take into account the individual state of beta-cell dysfunction. During T2DM disease progression, intact proinsulin values increase after a meal challenge, reflecting the inability of the beta-cells to adequately intracellularly cleave intact proinsulin into insulin and C-peptide on demand. At late-stage disease, the “exhausted” beta-cells produce only low levels of intact proinsulin anymore and therefore also low levels of insulin/C-peptide.

The main demographic and baseline characteristics are summarized in Table 2. BMI, HbA1c and sex distribution are depicted in supplementary figure S1 and provided in the supplementary data file (worksheet “participants_characteristics”). On average, healthy subjects were leaner with mean BMI equal to 23.7 (± 3.5) compared to 34.3 (± 5.0) and 31.5 (± 4.7) in pre-diabetic and T2DM groups, respectively. The groups were different in terms of gender distribution. All T2DM patients were male while 70% of healthy subjects were female.

**Table 2:** Demographics and baseline characteristics of the study population.

	Healthy (N = 10)	Pre-diabetic (N = 10)	T2DM (N = 10)
Age (years)	46.4 (12.7)	52.3 (8.3)	55.0 (7.9)
Age range	26–60	42–64	37–64
Sex = Male	3 (30%)	6 (60%)	10 (100%)
Sex = Female	7 (70%)	4 (40%)	0
Caucasian/White	10 (100%)	10 (100%)	10 (100%)
Weight (kg)	65.8 (13.2)	102.3 (26.4)	101.0 (16.9)
Weight range (kg)	50.3–84.5	69.6–149.6	75.0–123.0
Height (cm)	165.9 (6.6)	171.1 (11.4)	178.8 (7.8)
BMI (kg/m^2^)	23.8 (3.6)	34.4 (5.0)	31.5 (4.8)
BMI range (kg/m^2^)	20.1–28.9	28.2–41.4	26.4–38.9

### Serum metabolome: results summary

**Figure 3:**
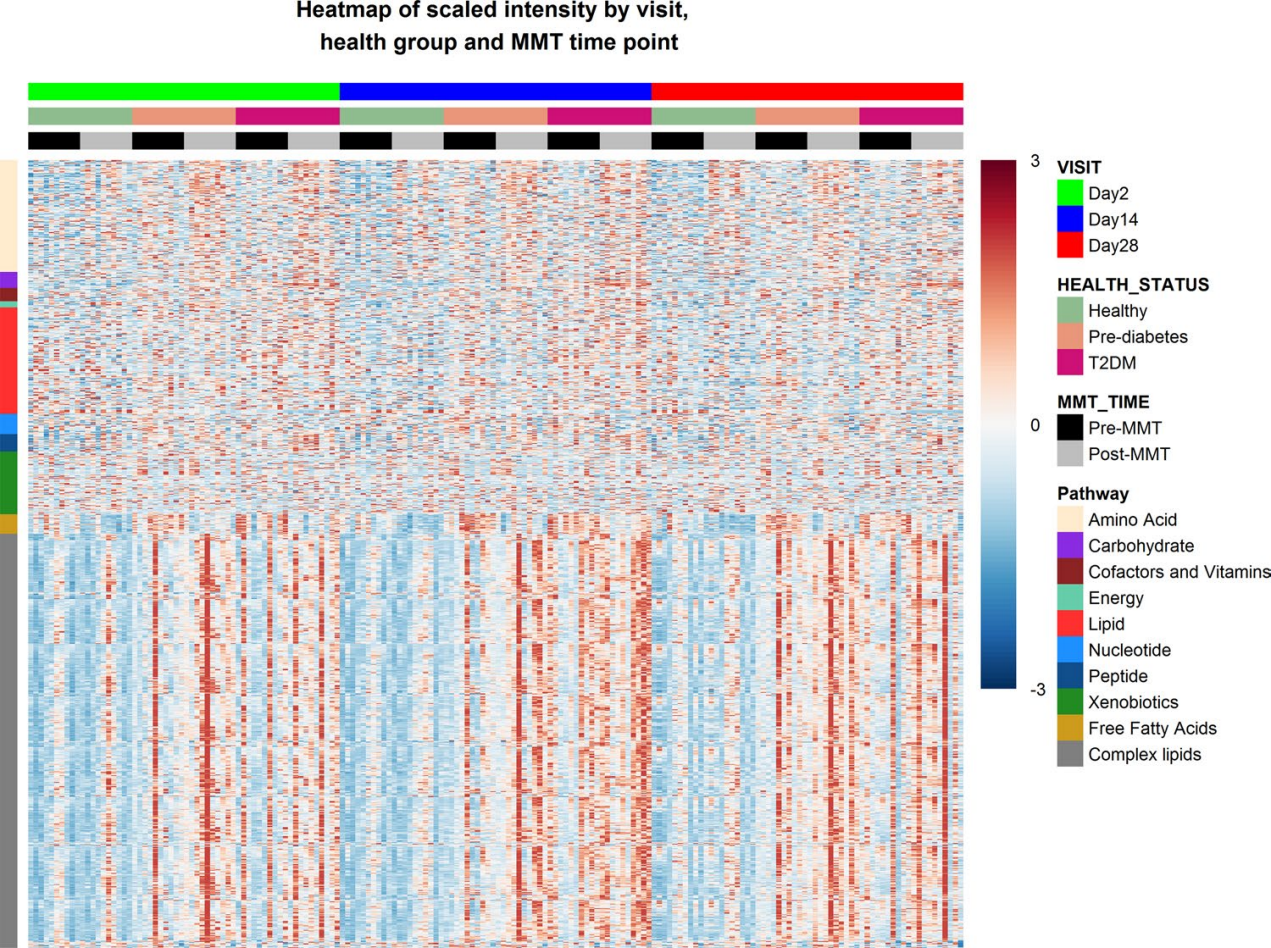
Heat map representing serum metabolite levels for each individual study participant (*horizontal*) ordered by study day, subject category and nutritional status, and individual metabolite (*vertical*) ordered by metabolic pathway.

Relative levels of all detected metabolites across the three study visits, before and after a standardized mixed meal and across the three metabolic health groups are provided in the supplementary data file and depicted as a heat map in Fig. 3. A repetitive pattern was observed comparing the map across visits, indicating overall reproducibility of metabolite levels and changes in response to meal intake on each of the three in-house study days. Differences in changes of metabolites in response to meal intake can be observed for different pathways: Whereas there is an increase in, e.g., amino acid levels in response to meal intake, free fatty acids were found to decrease after the mixed meal in all three health groups. Notably, however, there were higher prandial amino acid and fasting free fatty acid levels in T2DM patients and pre-diabetic individuals compared to healthy subjects. This reflects insulin resistance and the resulting lack of post-prandial suppression of lipolysis and reduced amino acid uptake in pre-diabetic and diabetic individuals. Large inter-individual variability is seen, e.g., for complex lipids that are exceptionally high in some pre-diabetic and diabetic individuals. However, these patterns are consistent across the three study visits, thus indicating low intra-individual variability over time (see below).

### Serum metabolome: principal component analysis (PCA)

**Figure 4:**
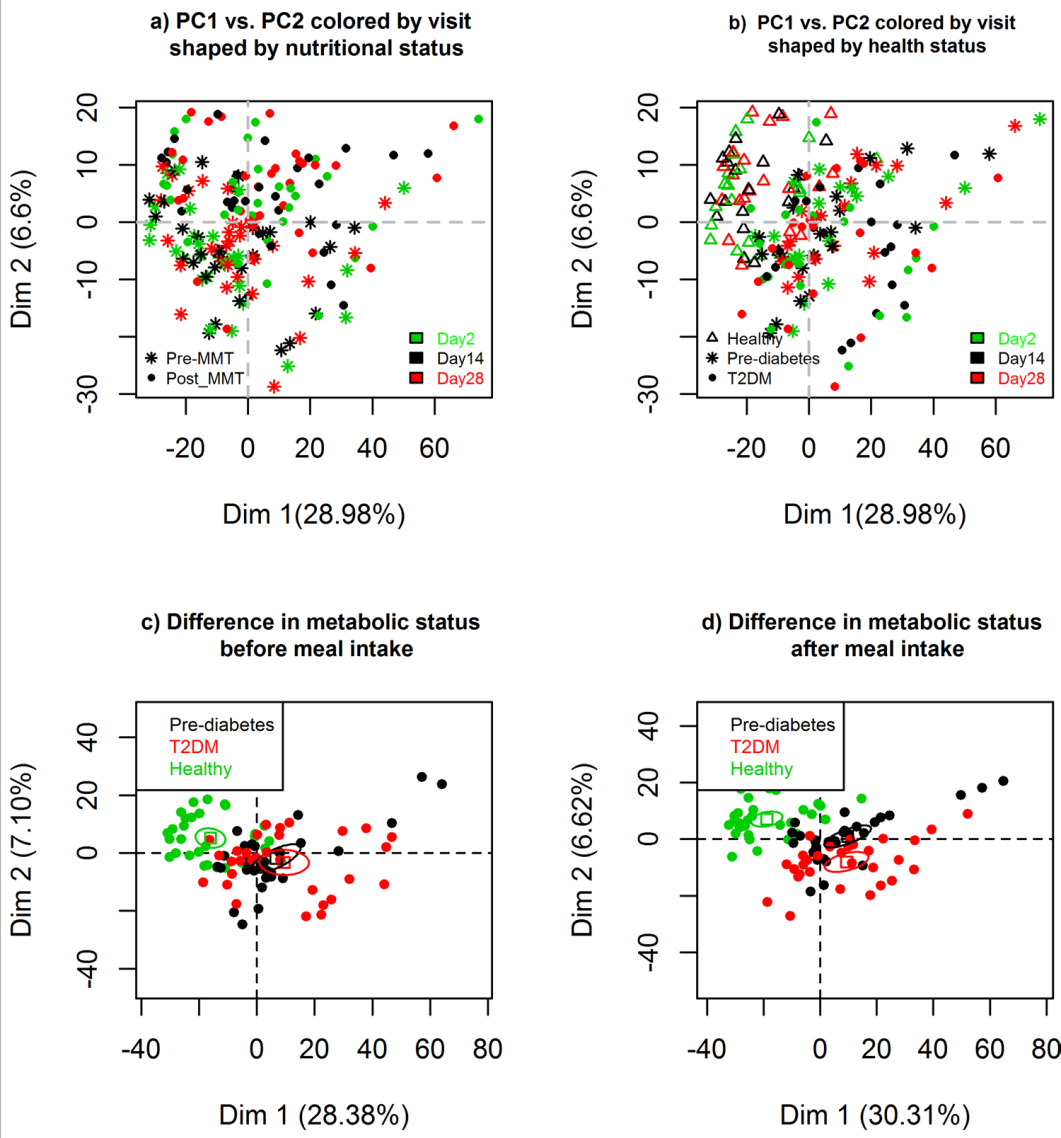
Principal component analyses for differences in metabolite levels between study days (**a**, **b**) or health groups (**c**, **d**). Colors and symbols refer to different study days, health groups or nutritional status as described in the diagrams.

The first two principal components of the serum metabolome containing 1438 individual metabolites are depicted in Fig. 4, each point representing one sample. Out of the different metabolite classes, PCA dimension 1 correlated strongly with triacylglycerides (TAG) and diacylglycerides (DAG) whereas dimension 2 was primarily determined by free fatty acids (FFA, negative correlation), lysophosphatidylethanolamines (LPE) and lysophosphatidylcholines (LPC, supplementary table S1).

There is a clear overlap between samples from different study visits, indicating a global consistency of metabolite levels over time (Fig. 4a,b). In Fig. 4a, the shapes of the symbols correspond to nutritional status (pre-MMT or post-MMT). As expected, there is a marked difference between pre- and post-meal levels, being mainly driven by the second principal component. Figure 4b shows the PCA with the shapes of the symbols corresponding to different health groups. There was a clear difference between metabolite levels in healthy subjects compared to pre-diabetic individuals and T2DM patients with most of the pre-diabetic subjects being in between the healthy and T2DM groups. Differences were mainly driven by the first principal component highly correlated with triacylglycerides (TAG). These results suggest that TAGs were increased in T2DM patients compared to pre-diabetic and healthy subjects. The data set was further split into pre-MMT and post-MMT sub data sets and the PCA was computed for both data sets separately. Figure 4c shows a clear separation of healthy from pre-diabetic and T2DM samples before MMT with the diabetic and pre-diabetic groups strongly overlapping. In contrast, after meal intake, pre-diabetic individuals differ from T2DM patients along the second principal component as illustrated by the separate confidence ellipses (Fig. 4d). Thus, subjects can be better stratified on their metabolic status after a meal intake compared to pre-meal stratification. The three black dots on the upper right (Figs. 4c,d) correspond to one pre-diabetic subjects that showed elevated C-peptide level at screening (supplementary figure S2). The three black dots more distant from the other black dots (Fig. 4d) correspond to one pre-diabetic subject that was however, similar to the other pre-diabetic subjects in term of screening parameters.

### Intra-individual variability of the serum metabolome: Intra-class correlation coefficients

**Figure 5:**
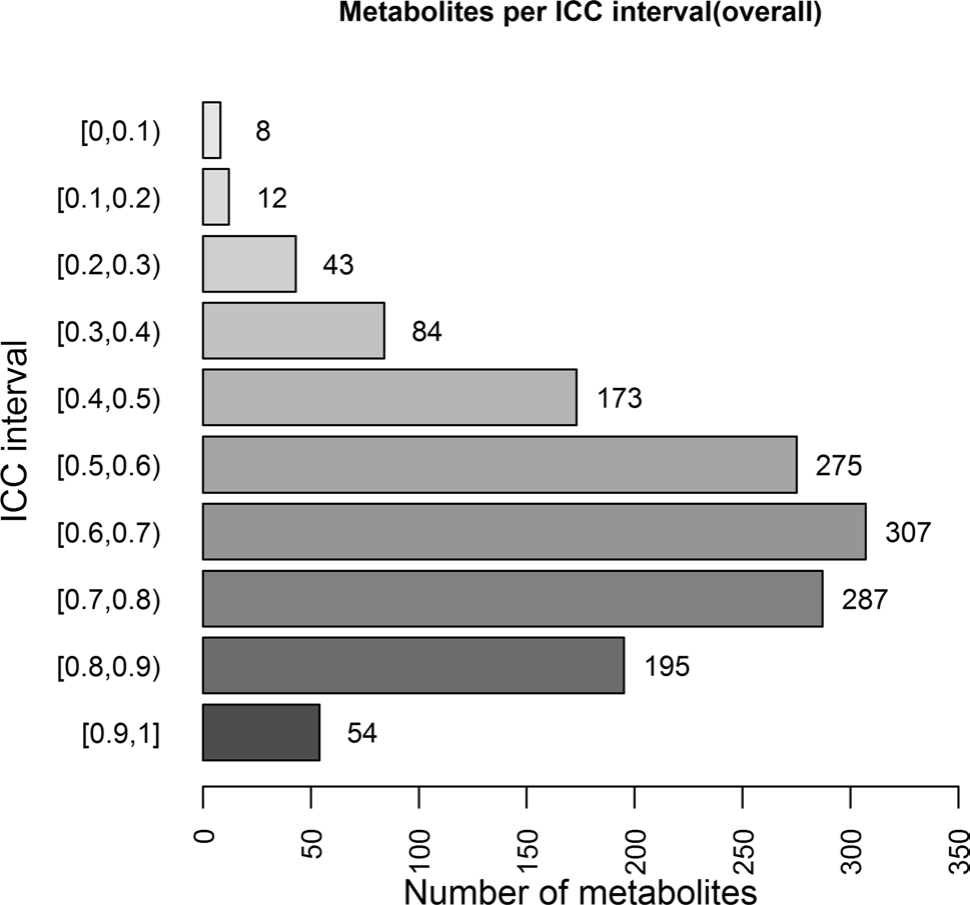
Histogram showing the number of individual metabolites per ICC interval.

The primary objective of our study was to investigate the intra-individual variability of the human serum metabolome in repeated measurements over a period of 4 weeks. To quantify the extent of intra-individual variability, intra-class correlation coefficients (ICC) were computed for each metabolite using Eq. 1. Of note, metabolites with low ICC (≤ 0.4) are perceived as highly variable or unstable over time. It means that the variation between time points was larger than the variation between individuals. For metabolites with higher ICC values, stability or reproducibility can be rated as fair (0.4–0.5), good (0.51–0.74) or excellent (0.75 and above)^8,9^. 1291 out of 1438 metabolites had an ICC above 0.4, corresponding to 90% of metabolites that can be considered as at least fairly stable over time. 1118 metabolites (78%) showed at least good stability (ICC > 0.5), and 407 (28%) metabolites showed excellent stability (ICC of 0.75 or higher). The mean (± SD) and median ICCs across all metabolites were determined as 0.63(0.17) and 0.65, respectively. These results show a fundamental stability of the human serum metabolome suggesting that the majority of metabolites can be good potential biomarkers. The number of metabolites per ICC interval is provided in Fig. 5.

**Figure 6:**
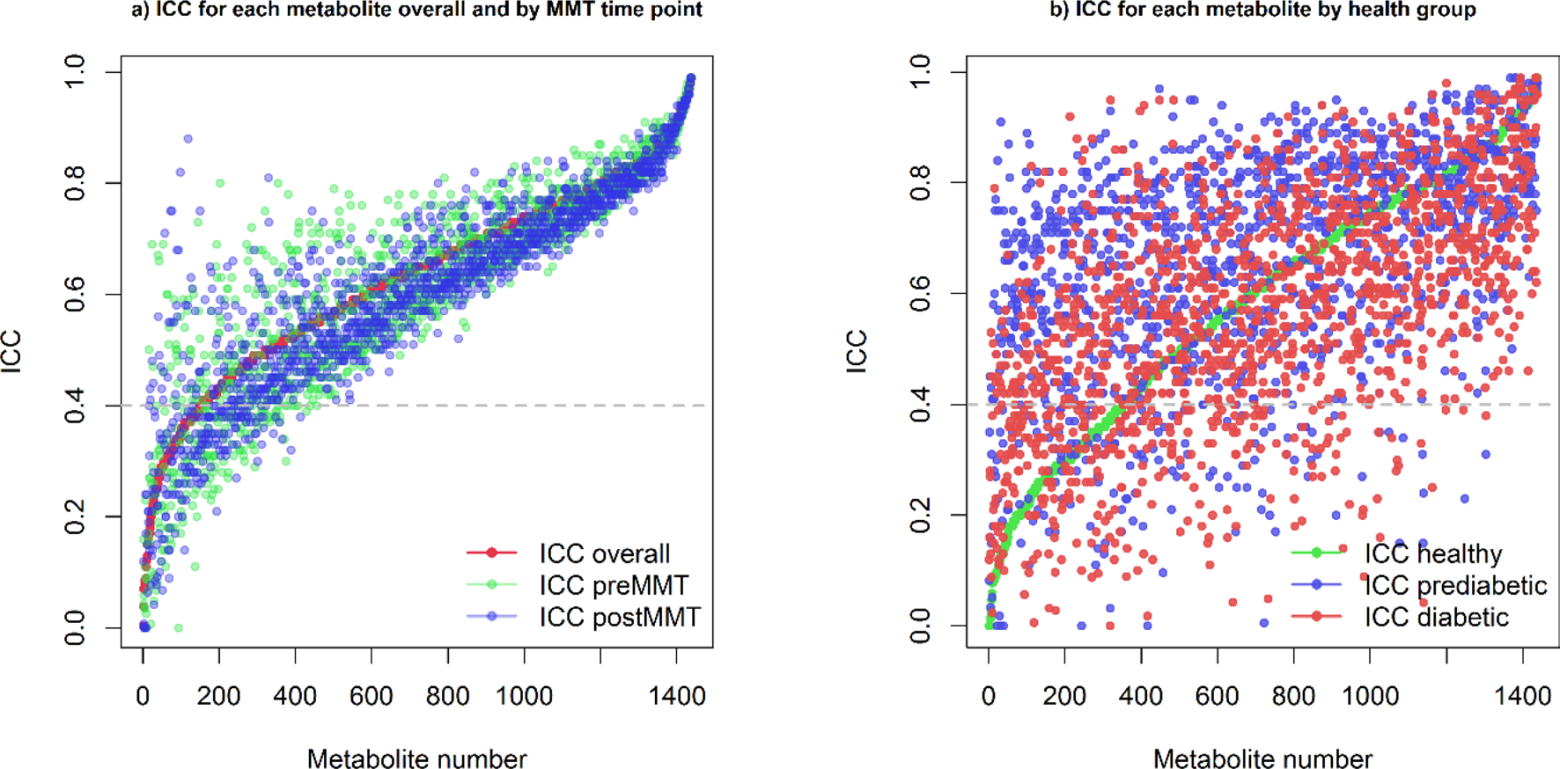
Distribution of ICCs by (**a**) nutritional status and (**b**) health group. In (**a**), metabolites are ranked by their ICC across all measurements (red), ICCs for the same metabolites under fasting or fed conditions are shown in green and blue, respectively. In (**b**), metabolites are ranked by their ICCs in the group of healthy individuals (green). ICCs for the same metabolites in the groups of pre-diabetic individuals and patients with T2DM are shown in blue and red, respectively.

We also tested if the variance of metabolite abundance depends on metabolic condition or nutritional status by splitting the data and computing the ICC in sub-data sets. The ICC values for pre-MMT samples were comparable to that of post-MMT samples and global ICC values (Fig. 6). There were some differences in intra-individual variability as defined by ICC between the different health groups (Fig. 6b) that could not be readily associated with specific metabolic pathways as the mean ICC was similar for each pathway across the three health groups (Table 3).

**Table 3:** Summary statistics for ICC by pathway.

Pathway	ICC	ICC healthy	ICC pre-diabetes	ICC T2DM	ICC pre-MMT	ICC post-MMT
All	0.63(0.17)	0.58(0.23)	0.66(0.2)	0.57(0.21)	0.61(0.19)	0.61(0.18)
Amino Acid	0.64(0.16)	0.6(0.21)	0.64(0.22)	0.6(0.22)	0.63(0.18)	0.63(0.17)
Carbohydrate	0.52(0.22)	0.46(0.23)	0.48(0.26)	0.55(0.23)	0.53(0.22)	0.52(0.2)
Cofactors and Vitamins	0.72(0.16)	0.65(0.26)	0.74(0.15)	0.66(0.14)	0.67(0.23)	0.71(0.15)
Energy	0.55(0.08)	0.46(0.13)	0.6(0.13)	0.56(0.09)	0.58(0.09)	0.54(0.13)
Lipid	0.56(0.24)	0.52(0.24)	0.61(0.26)	0.52(0.27)	0.56(0.25)	0.56(0.24)
Lipid/steroid	**0.83(0.22)**	**0.77(0.22)**	**0.83(0.19)**	**0.89(0.12)**	**0.81(0.26)**	**0.81(0.26)**
Neutral Complex Lipids^a^	0.65(0.14)	0.59(0.24)	0.71(0.14)	0.56(0.18)	0.64(0.15)	0.62(0.15)
Nucleotide	0.58(0.21)	0.56(0.24)	0.56(0.23)	0.59(0.23)	0.55(0.23)	0.57(0.23)
Peptide	0.63(0.18)	0.63(0.2)	0.59(0.21)	0.61(0.18)	0.6(0.21)	0.65(0.19)
Phospholipids	0.61(0.13)	0.61(0.16)	0.6(0.19)	0.56(0.17)	0.6(0.14)	0.56(0.15)
Sphingolipids	0.71(0.17)	0.66(0.23)	0.76(0.2)	0.59(0.19)	0.67(0.2)	0.7(0.18)
Xenobiotics	0.55(0.19)	0.48(0.22)	0.59(0.21)	0.52(0.21)	0.49(0.23)	0.5(0.22)

^a^Cholesterol esters, diacyglycerols, free fatty acids, triacylglycerols, ceramides, dihydroceramides.

Individual metabolites with the 10 highest and 10 lowest ICC values are summarized in supplementary table S2. Interestingly, among the top 25 metabolites, there are 14 steroids indicating tight control and stability of this class of metabolites. This is also reflected in the high mean ICC of 0.83 across all members of the steroid class (Table 3, in bold).

**Figure 7:**
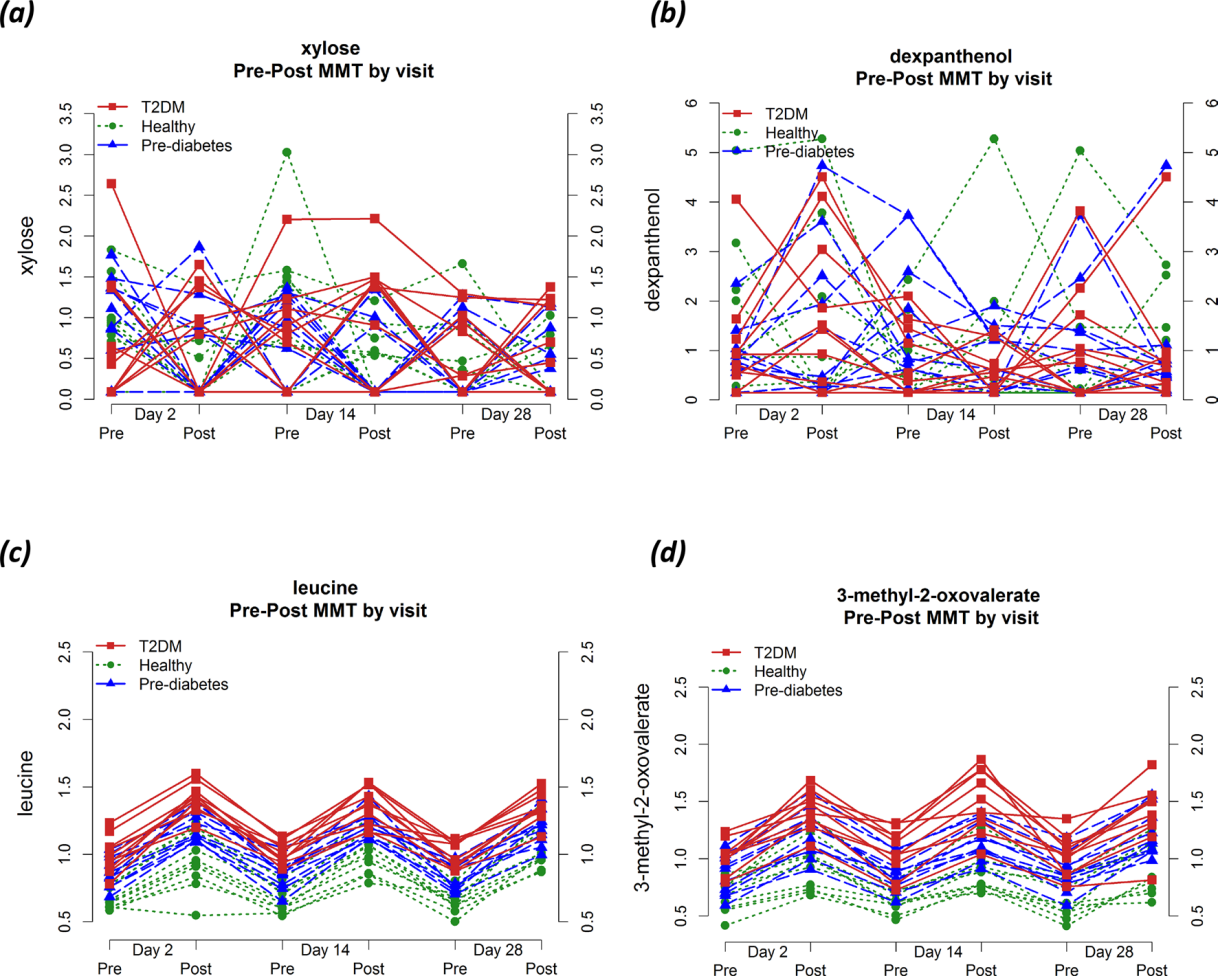
Examples of unstable (**a**, **b**) and stable (**c**, d) metabolites colored by health group.

Illustrative examples of unstable and stable metabolites are presented in Fig. 7: Xylose and dexpanthenol had ICC values of 0.09 and 0.11, leucine and 3-methyl-2-oxovalerate had ICC values of 0.84 and 0.7, respectively.

ICC by pathway was computed by summarizing the ICC values for all metabolites in that pathway. All metabolic pathways showed longitudinal stability with an average ICC above 0.4 regardless of health group and nutritional status (see Table 3). Although there were no obvious differences between major metabolic pathways, some subclasses such as steroids (ICC = 0.83 ± 0.22) or free fatty acids (ICC = 0.44 ± 0.14), respectively, show less or more intra-individual variability over time.

### Visit-to visit variability

Metabolites were globally very stable between visits as observed with the ICC and PCA. Only cytidine levels were significantly different (FDR < 0.05) with a fold-change above 1.5 between day 14 and day 28, while 9 metabolites were tested as significantly different with a fold-change > 1.5 between study days 2 and 28 (supplementary figure S3and supplementary table S3).

### Differences between health groups

The secondary objective of our study was to identify metabolite signatures of pre-diabetes and T2DM. As expected, there were considerable statistically significant differences in specific groups of metabolites between the healthy group and the pre-diabetic and T2DM groups both before and after the mixed meal. Pre-diabetic and T2DM groups were more overlapping suggesting similar metabolic profiles for these groups.

#### Difference before MMT

**Figure 8:**
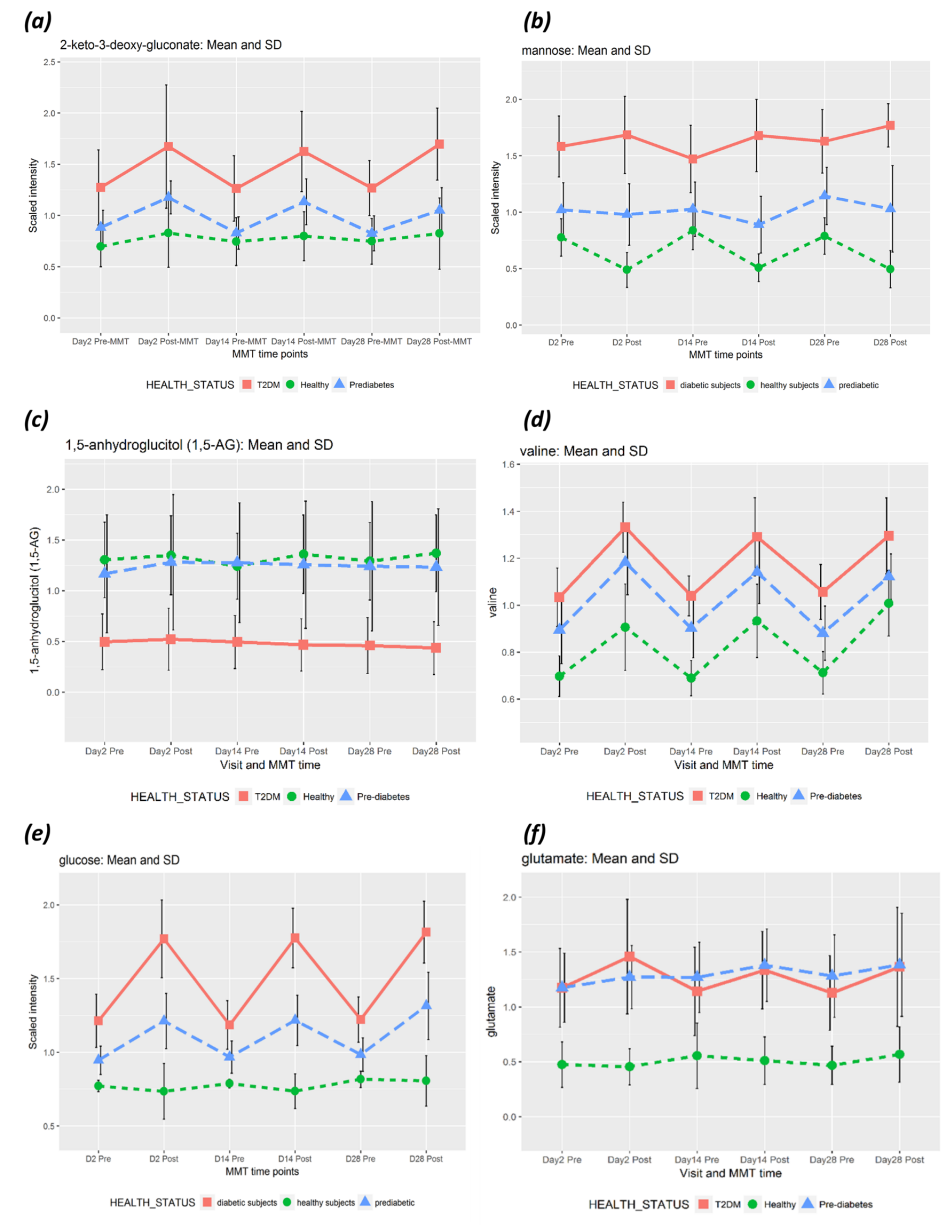
Selected metabolites differing between health groups before the mixed-meal test. Diagrams show mean metabolite levels per health group ± SD for study days 2, 14 and 28, pre- and post-MMTT for (**a**) 2-keto-3-deoxy-gluconate, (**b**) mannose, (**c**) 1,5-anhydroglucitol, (**d**) valine, (*i*) glucose and (**f**) glutamate.

49 out of 1438 metabolites were significantly different between healthy subjects and T2DM patients before meal intake. For example, carbohydrates (e.g., glucose, mannose, fructose), branched-chain amino acids (BCAA, e.g., leucine [Fig. 7c], valine [Fig. 8d], isoleucine), intermediates of BCAA metabolism (e.g., 3-methyl-2-oxovalerate [Fig. 7d], glutamate, gamma-glutamyl, alpha-hydroxyisocaproate and beta-hydroxyisovalerate), and free fatty acids (e.g., 18:0, 24:1, 22:1) were significantly higher in T2DM patients compared to healthy subjects. 1,5-anhydroglucitol (1,5-AG) was elevated in healthy subjects compared to T2DM before MMT but also post MMT. Of note, there was no difference in 1,5-AG levels between the groups of healthy and pre-diabetics individually, and 1,5-AG did not change upon meal intake (Fig. 8c). 1,5-AG levels were independent from BMI per group and well separated by HbA1c (supplementary figure S4).

32 out of 1438 (2.2%) metabolites were significantly different between healthy and pre-diabetic subjects before MMT with an FDR < 0.05. Glutamate (Fig. 8f) and cystine were elevated in pre-diabetic subjects before but also after MMT intake. Only 7 metabolites out of 1438 were significantly different between pre-diabetic subjects and T2DM patients before MMT with an FDR < 0.05. The known metabolites demonstrating significant differences were mannose, glucose, glycerophosphorylcholine (GPC), sphingomyelin(18:1), 3-hydroxydecanoate and 2-keto-3-deoxy-gluconate. Examples of metabolites differing between health groups are shown in Fig. 8.

#### Differences post-MMT

As illustrated in Fig. 4d, differences between health groups became larger 1 h after intake of a defined mixed meal: 6.5% of all metabolites were significantly different between healthy and T2DM patients 1 h after the MMT with an FDR < 0.05. For example, members of the phospholipid metabolism pathways (e.g., Arachidonoylcholine, docosahexaenoylcholine and dihomo-linolenoyl-choline) were decreased in T2DM patients after MMT compared to healthy subjects. 14 metabolites were significantly different between healthy and pre-diabetic individuals after MMT with an FDR < 0.05. 11 metabolites were significantly different between pre-diabetic subjects and T2DM patients after MMT with an FDR < 0.05: Glucose, mannose, 3-methylglutaconate, 3-hydroxyoctanoate, fructose, 2-keto-3-deoxy-gluconate, glycodeoxycholate, 3-hydroxydecanoate, glycolithocholate sulfate, sphingomyelin(18:1) and 1,5-anhydroglucitol (1,5-AG).

#### Difference in MMT response between health groups

**Figure 9:**
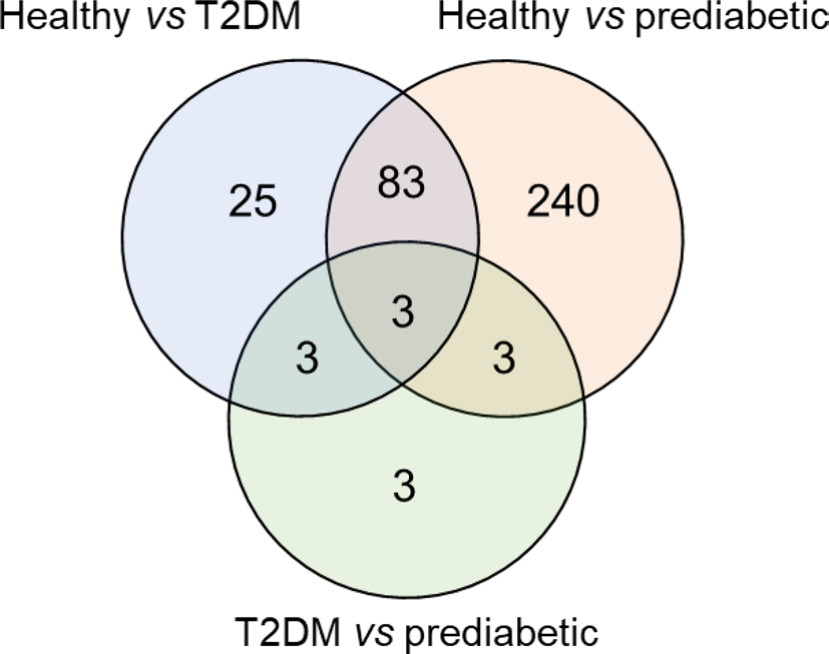
Venn diagram showing number and overlap of metabolites with significant differences in the response to MMT.

The MMT response, i.e. the change in metabolite levels post-MMT vs. pre-MMT, was compared between healthy subjects, pre-diabetic individuals and T2DM patients. Results are summarized in Fig. 9 that shows the overlap of metabolites with pairwise differences in changes in response to meal intake between different health groups. The three metabolites in the overall intersection were glucose, mannose and 3-methylglutaconate, an intermediate of leucine metabolism.

## Discussion

The primary objective of our study was to interrogate the intra-individual variability of the human serum metabolome over a comparably short period of time in individuals with different metabolic health and under fasting and fed conditions. An additional goal was to identify metabolic signatures differentiating metabolically healthy individuals from subjects with impaired glucose tolerance (IGT) or T2DM. For these purposes, we performed a 4-week study to collect blood samples at three different days following an overnight fast and a defined mixed meal, followed by a global serum metabolomics and lipidomics analysis. Study participants included healthy subjects, individuals with IGT and patients with T2DM (n = 10 each).

The ICC was high for of the majority of metabolites suggesting that metabolites were stable during the study with almost no visit effect. In addition, exploratory statistical tests for visit-to-visit difference were not significant.

This is, to our knowledge, the first and most extensive study investigating serum metabolome variability in human subjects with different metabolic health conditions over a period of 4 weeks in a tightly controlled dietary environment of a single inpatient clinical center. Our results show that the human serum metabolome is remarkably stable when measured repetitively, and that low variability is seen independent of nutritional and metabolic health status. Previous studies investigating the temporal variability of the circulating metabolome include the study by Kim et al.^4^, who came to similar conclusions regarding the stability of the metabolome before and after intake of a meal. However, their results were based on samples taken on three consecutive days, focused on healthy individuals and patients with polycystic kidney disease and was limited to 121 plasma metabolites. In another study, Zheng et al.^10^ investigated the medium-term variability of the human serum metabolome of participants in the ARIC study, finding an ICC across all metabolites investigated of 0.6 and 82% of metabolites having an ICC >  = 0.4, very similar to our findings. However, their analysis was based on only two samples per subject taken 4–6 weeks apart from each other under fasting conditions and limited to 178 metabolites. A median ICC of 0.57 was reported in^8^ for fasting serum samples taken approximately 4 months apart, based on the analysis of 163 metabolites. Recently, based on repeated measurements in the Netherlands Epidemiology of Obesity Study, median ICC values of 0.72 and 0.62 for fasting plasma and 0.66 and 0.64 for prandial plasma were reported based on ^1^H-NMR spectroscopy analysis of 148 metabolites in samples taken approximately 4 months or 3 years apart, respectively^9^. Finally, Yousri et al. also found a high degree of metabolite level conservation in 818 subjects of the KORA cohort when investigating 212 metabolites in samples that were collected up to 13 years apart from each other^11^.

There is a much larger body of studies available that interrogate the influence of dietary interventions or metabolic disease on the circulating metabolome (reviewed, e.g., in^12^). A landmark study using multiplatform metabolomics identified a metabolic footprint of T2DM comparing 40 individuals with T2DM and 60 healthy controls from a population-based study^13^ using samples taken under fasting conditions. Of note, key metabolites associated with T2DM identified in their study, like BCAA, mannose, 1,5-AG, ketone bodies or medium chain-length free fatty acids, could be confirmed in our study. In addition, our study showed that 1,5-AG is independent of nutritional status and lower in T2DM but not pre-diabetes, indicating an association with fasting but not prandial glucose (see Fig. 8c). Of note, three metabolite biomarkers (glycine, LPC 18:2, acetylcarnitine) for prediabetes that were recently identified and confirmed in the KORA and EPIC-Potsdam cohorts^14^, respectively, could also be confirmed in our study (supplementary figure S5).

Likewise, BCAA, branched-chain ketoacids (BCKA) and their catabolic derivatives like glutamate or acylcarnitines were found to be strongly associated with IGT and T2DM, as previously described in multiple studies^15–22^ (see Figs. 7c,d; 9d,f.). Thus, previous results could be corroborated even though the number of study participants was small and the identification of metabolites to differentiate between the three health groups was not the primary objective of our study (see below).

Major strengths of our study are (1) the tight control of nutritional status and sampling time. In addition, whereas most of the previous studies investigating the variability of the human metabolome relied on samples taken at two different time points in healthy volunteers, our results are based on six measurements at three time points in three groups of individuals with different metabolic health and before and after intake of a defined mixed meal. (2) The number of detected metabolites across a wide range of biochemical pathways: With 1438 metabolites, 1202 being assigned to known structures, included in our final analysis, this is one of the largest untargeted metabolomics approach investigating intra-individual variability using multiple samples per subject. (3) Dependence on metabolic health and nutritional status: We could demonstrate that intra-individual variability of the serum metabolome is largely independent of nutritional and metabolic disease status. Furthermore, by including a group of individuals with impaired glucose tolerance but without T2DM, we could identify metabolites that differ between this group and healthy subjects on one hand and patients with T2DM on the other or may respond differently to intake of a mixed meal. (4) Diversity of the study population: The study participants cover a broad range of age and weight/BMI which helps to generalize conclusions regarding intra-individual variability of the metabolome (but may negatively impact comparison between different health groups, see below). (5) Robustness of results: We have confirmed multiple previously identified metabolites associated with meal intake or metabolic disease. (6) Multitude of samples collected under tightly controlled conditions: The study has generated numerous additional samples at various time points following the mixed meal, in different matrices (serum, EDTA-, Li-heparin-, NaF- and p800-plasma) and body fluids (including urine) that are available for additional analyses beyond the metabolomics investigation described here.

Limitations of our studies are the following: (1) The metabolomics method applied in our study covers a large number of metabolites but provides relative levels rather than absolute concentrations. While this is not necessarily relevant for the investigation of temporal variability, it means that for, e.g., the differentiation between study groups no absolute threshold values can be defined. (2) Diversity of the study population: Participants with pre-diabetes or T2DM were older and heavier than healthy controls, and there was a gender disparity especially in the T2DM group where only male participants were recruited. In addition, group sizes were small (n = 10 each), limited by resource constraints. Whereas for the investigation of intra-individual variability each participant served as his or her own control, thereby requiring smaller groups, the study was not powered to robustly determine inter-individual differences, e.g. between health groups Thus, differences in metabolites between the three health groups could be due to factors other than metabolic disease. With respect to the second objective of identifying metabolic signatures of pre-diabetes and T2DM, our study should therefore be seen as hypothesis-generating rather than providing definitive conclusions. (3) Variation in metabolite levels is a composite of biological and technical variation. While all efforts were made to limit technical variability through, e.g., multiple embedded QC standards in every sample and technical replicates across each study run day, it still may be a major contributor for at least a subset of metabolites.

In conclusion, we have comprehensively interrogated the intra-individual variability of the human serum metabolome over time and depending on metabolic health and nutritional status. The majority of metabolites were determined to be robust and stable in repeated measurements and could thus serve as potential biomarkers for conditions in which they are regulated. Furthermore, previously identified biomarkers for pre-diabetes or T2DM could be confirmed and were found to be stable over time. Samples (serum, EDTA-, Li-heparin-, NaF- and p800-plasma, urine) collected in our study from three different health groups over a period of 4 weeks and under fasting and fed conditions are available for further biomarker analyses.

## Methods

### Study conduct

The study was conducted in accordance with the guidelines of the 18th World Medical Assembly (Helsinki 1964) and all applicable amendments laid down, and the ICH guideline for Good Clinical Practice GCP, all applicable laws, rules and regulations. The study was approved by the local ethics committee (Ethik-Kommission der Bayerischen Landesärztekammer, Mühlbaurstraße 16, 81,677 München). Informed consent was obtained prior to the conduct of any study-related procedures. The subject informed consent form was prepared according to local regulations and requirements.

### Study population

This was a single-center biomarker collection study conducted at Nuvisan GmbH (Neu-Ulm, Germany). Apart from a standardized mixed meal, no study drug was applied or other therapeutic intervention performed. Out of 65 study participants enrolled and screened with an oral glucose tolerance test (OGTT), 30 subjects were assigned to the respective study groups: healthy, prediabetic or T2DM individuals (n = 10 each), depending on HbA1c as well as on fasting and OGTT-challenged glucose, C-peptide and intact proinsulin measurements, based on ADA criteria^7^. Detailed inclusion and exclusion criteria for the three health groups are provided in supplementary information. Individuals that could not unambiguously allocated to one of the three study groups were excluded from further study participation and from metabolomics analyses.

The study design is illustrated in Fig. 1. On study days 2, 14 and 28, participants provided biological samples longitudinally before and after a mixed meal test (MMT). The mixed meal consisted of a 400-ml drink (Ensure Plus, Abbot) containing 600 kcal and composed of 53.8% carbohydrates, 16.7% protein and 29.5% fat, and a standard protein bar as solid component. All 30 subjects completed the study without any serious adverse events due to mixed meal intake. There were three in-house days on Days 1–2, 13–14 and 27- 28 during which subjects stayed on site overnight and provided biological samples in fasted conditions the next morning before, during, and after an MMT. Two ambulatory biological sample collections were also performed at Day 7 and Day 21 but these samples were not analyzed using metabolomics as the conditions were not under full control of the investigators. Participants were asked not to change lifestyle or diet over the course of the study. No medication was allowed throughout the study with the exception of antihypertensive treatment (all classes) and medication for short-term treatment of mild clinical disorders such as intermittent headaches up to 48 h before screening or study visits. For the T2DM group, metformin was allowed at any dose regimen but had to be stopped the evening before OGTT or MMT.

The screening procedure included standard safety parameters (vital signs, clinical chemistry), measurement of glycosylated hemoglobin (HbA1c), fasting plasma glucose (FPG) and an OGTT. Before starting the OGTT, glucose values were determined via capillary blood sampling, and hyperglycemic values > 270 mg/dL blood glucose in T2DM patients led to postponing of OGTT or study exclusion, as a safety measure. The OGTT was carried in the morning after an overnight fast of at least 10 h (in-house visits), and after at least 3 days of unrestricted diet and unlimited physical activity.

### Sample collection

Serum (collected with blood collection tubes containing no coagulation activator) stood upright for at least 20–30 min before centrifugation to allow for coagulation to take place. Following centrifugation, the supernatant containing serum was split into aliquots of 200 μl volume, using appropriately labeled cryo storage tubes. Storage tubes were then transferred into storage racks and immediately frozen at − 80 °C. Samples were shipped on dry ice with a temperature tracker.

### Global metabolomics and lipidomics analysis

Serum samples were analyzed at Metabolon Inc. (Morrisville, NC, USA). Global metabolomics was performed as previously described^23–26^. Briefly, samples were extracted by agitation with methanol. Extracts were then split to enable analysis by four different methods. All methods used a Waters Acquity ultra-performance liquid chromatography (UPLC) system coupled to a Thermo Scientific Q-Exactive high resolution accurate-mass spectrometer. Raw data extraction, peak identification, and quality control processing were carried out using the Metabolon proprietary software. Metabolite identification was done through comparison with a library of chromatographic and MS data from authenticated standards. Metabolite abundances were determined by their area under the curve (AUC). Lipidomics was done as previously described^27^. Briefly, lipids were extracted from serum samples using a butanol:methanol (BUME) mixture (3:1) followed by two-phase extraction into heptane:ethyl acetate (3:1) using 1% acetic acid as buffer. Reconstituted extracts were infused into a SelexION equipped Sciex 5500 QTRAP. The scan was performed in multiple reaction monitoring (MRM) mode. Individual lipid species were quantified by referencing to known concentration of internal standard. Lipid class concentrations were calculated from the sum of all molecular species within a class, and fatty acid compositions were determined by calculating the proportion of each class comprised by individual fatty acids. Further experimental details and the analysis process are described in Supplementary Methods.

### Statistical analysis

#### Data pre-processing

Six serum samples were analyzed from each study participant: one sample taken after an overnight fast (t = –30 min, see Fig. 1), one sample 1 h after intake of a defined mixed meal, for a total of three times at study visit days 2, 14 and 28. Untargeted serum metabolomics and lipidomics were performed in two runs, with two batches in the first and four batches in the second run. Potential batch effects were adjusted for using the following procedure: A pool of small aliquots was made from all samples and was analyzed in both runs for normalizing the data and adjusting for batch effects. The data were generated for seven healthy subjects in the first run. Here, 1637 known metabolites and 315 unnamed metabolites were identified, leading to a total of 1952 metabolites detected. In the second run, data were generated for the remaining 23 participants (3 healthy subjects, 10 pre-diabetic subjects and 10 T2DM patients). Here, 1391 known metabolites and 305 unnamed metabolites were identified leading to a total of 1696 metabolites detected. The two data sets were merged utilizing the reference data generated in both study parts. The merged data set consisted of 1486 metabolites with a very good detection rate: 1064 were identified in all samples; 213 metabolites had the proportion of values below the lower limit of quantification (LLOQ) in the interval (0.10%]; 161 metabolites had the proportion of values below LLOQ between 10 and 50% of all samples and only 48 metabolites had more than 50% of LLOQ. The raw data were scaled for median metabolite equal to one. For metabolites with less than 50% of values below LLOQ, values below LLOQ were imputed by the minimum of detected values across all samples. Metabolites with more than 50% of values below LLOQ were discarded from all statistical analyses. Outliers were determined as values outside of the range the [Q1 − 3*IQR, Q3 + 3*IQR], where Q1 denoted the 25% percentile of the metabolite values across all samples, Q3 the 75% percentile and IQR the interquartile range. Those values are extreme outliers based on the Tukey method and were imputed by Q3 + 3*IQR and Q1 − 3*IQR, respectively. No log-transformation was applied after scrutinizing the data for Gaussian distribution.

#### Longitudinal stability of metabolites

##### Principal component analysis (PCA)

The PCA was computed with all metabolites to reduce the dimensionality of the data while retaining maximal variability. Each sample was projected on the first two principal components and was colored or shaped by sub-group or visit for visualizing global visit-to-visit variability, the change due to meal intake and the differences due to metabolic status.

##### Intraclass correlation coefficient (ICC)

For each metabolite, the ratio of variability between individuals and the variability between sampling time points was estimated with the following linear mixed model. For a given metabolite, measurement for time point j and subject k was modeled with:

Equation 1: model for computing the ICC

1 $$ {\text{y}}_{{{\text{jk}}}} =\upmu + {\text{Sub}}_{{{\text{jk}}}} + {\text{ MMTT }} + {\text{HealthStatus}} + {\text{MMT:HealthStatus}} +\upvarepsilon _{{{\text{jk}}}} $$

ICC is the ratio of inter-subject variance and total variance, it has been estimated with:

$$ICC = \frac{{\sigma_{Subj}^{2} }}{{\sigma_{Total}^{2} }}$$.

ICC around 0 means low correlation between observations for the same subject, whereas ICC around 1 means high correlation between observations for the same subject^28^. The ICC for each metabolic pathway was derived as mean and standard of ICCs of metabolites in this pathway. A single metabolite or metabolic pathway with an ICC value below 0.4 was considered as poorly stable. Ratings of stability for metabolites with ICC values above 0.4 were as follows: 0.4–0.5: moderate, 0.51–0.74: good, 0.75 or higher: excellent^8,9^.

##### Pathway stability using median polish

An additive model (two way decomposition) using Tukey's median polish procedure was computed to derive a composite marker from all metabolites in each pathway (Amino Acid (163), Carbohydrate (22), Cofactors and Vitamins (18), Energy (8), Lipid (161), Neutral Complex Lipids (605), Nucleotide (29), Peptide (22), Phospholipids (210), Sphingolipids (54) and Xenobiotics (97)). The composite biomarkers were explored for longitudinal variability of pathways.

##### Test statistics for visit, MMT and metabolic status effect on metabolites

For identifying metabolites significantly altered by the metabolic status, unstable metabolites across study visits and metabolites that changed according to the fasting or feeding condition, a linear mixed model was computed (see Eq. 2).

Equation 2: linear mixed model for all subgroup differences

2 $$ {\text{Metabolite}} = {\upmu } + {\text{Visit}} + {\text{health}}\_{\text{group}} + {\text{MMTT}} + {\text{batch }} + {\text{MMT}}*{\text{health}}\_{\text{group}} + \left( {1|{\text{Subject}}} \right) +\upvarepsilon $$

where Visit represents a factor variable with levels Day2, Day14 and Day28; health_group (Healthy, pre-diabetic, T2DM groups); MMTT (pre-MMTT and post-MMTT); batch (6 modalities); MMT*HealthStatus the interaction between MMTT and health_group were fixed effects and subject as random effect. Day2, pre-MMTT healthy and the first batch in the pilot study were set as reference subgroups for the corresponding variables. All p-values were adjusted for the multiplicity to control the false discovery rate (FDR). The Benjamini–Hochberg procedure^29^ was used for this purpose.

### Software

The statistical analyses were performed using R version 3.2.3 (2015–12-10). In addition to the base package, we used the packages FactoMiner for PCA, pheatmap and RcolorBrewer for generating heatmaps, ggplot2, openxlsx, VennDiagram, and the packages lme4 and multcomp for linear mixed model and group comparisons.

## Supplementary information

Supplementary Information 1.

Supplementary Information 2.
