# Supplementary material for: Short-term variability of the human serum metabolome depending on nutritional and metabolic health status
Source: Sci Rep. 2020 Oct 1;10:16310. doi: 10.1038/s41598-020-72914-7 (PMC7530737; doi:10.1038/s41598-020-72914-7)
Supplement: Supplementary file 2 — Supplementary Information 2. [file 41598_2020_72914_MOESM2_ESM.docx]

**Supplementary Information to**

**Short-term variability of the human serum metabolome depending on nutritional and metabolic health status**

**Inoncent Agueusop^1^, Petra B. Musholt^1^, Beate Klaus^2^, Kendra Hightower^3^, Aimo Kannt^1,4,5*^**

^1^ Sanofi Research and Development, Frankfurt, Germany

^2^ Nuvisan GmbH, Neu-Ulm, Germany

^3^ Metabolon Inc., Morrisville, US

^4^ Experimental Pharmacology, Medical Faculty Mannheim, University of Heidelberg, Mannheim, Germany

^5^ Fraunhofer Institute for Molecular Biology and Applied Ecology, Division of Translational Medicine and Pharmacology, Frankfurt, Germany

*Corresponding author: aimo.kannt@web.de

**Overview**

- **Supplementary Tables S1-S3**
- **Supplementary Figures S1-S5**
- **Supplementary Methods**
  1. Study Inclusion and Exclusion Criteria
  2. Global Metabolomics Methods
- **References**

**Supplementary Tables**

**Supplementary table S1: Correlation of prinicipal components with metabolite classes**

| **Metabolite class** | **Correlation coefficient with Dimension 1** | **Correlation coefficient with Dimension 2** |
| --- | --- | --- |
| Total triacylglycerides (TAG) | 0.96 | -0.05 |
| Total diacylglycerides (DAG) | 0.92 | -0.13 |
| Total dihydroceramides (DCER) | 0.62 | -0.17 |
| Total ceramides (CER) | 0.58 | 0.071 |
| Total phosphatidylethanolamines (PE) | 0.56 | -0.011 |
| Total phosphatidylcholines (PC) | 0.37 | 0.26 |
| Total phosphatidylinositides (PI) | 0.37 | -0.18 |
| Total cholesteryl acids (CE) | 0.31 | 0.2 |
| Total free fatty acids (FFA) | 0.23 | -0.76 |
| Total sphingomyelins (SM) | 0.13 | 0.29 |
| Total lysophosphatidylethamolamines (LPE) | -0.092 | 0.61 |
| Total hexosylceramides (HCER) | -0.16 | 0.32 |
| Total lysophosphatidylcholines (LPC) | -0.29 | 0.52 |
| Total lactosylceramides (LCER) | -0.44 | 0.24 |
|  |  |  |

Supplementary Table S2: Metabolites with largest ICC and metabolites lowest ICC

| **Metabolites** | **ICC** | **ICC healthy** | **ICC pre-diabetic** | **ICC T2DM** | **ICC pre MMT** | **ICC post MMT** |
| --- | --- | --- | --- | --- | --- | --- |
| 4-hydroxychlorothalonil | 0.99 | 0.98 | 0.99 | 0.99 | 0.99 | 0.99 |
| pyroglutamine* | 0.98 | 0.99 | 0.97 | 0.96 | 0.98 | 0.98 |
| dehydroisoandrosterone sulfate (DHEA-S) | 0.97 | 0.97 | 0.98 | 0.97 | 0.97 | 0.96 |
| 4-androsten-3beta.17beta-diol disulfate (1) | 0.97 | 0.93 | 0.99 | 0.95 | 0.96 | 0.97 |
| 4-androsten-3beta.17beta-diol monosulfate (2) | 0.97 | 0.92 | 0.99 | 0.87 | 0.96 | 0.97 |
| 5alpha-androstan-3beta.17beta-diol disulfate | 0.96 | 0.97 | 0.95 | 0.97 | 0.96 | 0.94 |
| 4-androsten-3alpha.17alpha-diol monosulfate (3) | 0.96 | 0.95 | 0.98 | 0.95 | 0.96 | 0.95 |
| androsterone sulfate | 0.95 | 0.92 | 0.99 | 0.97 | 0.95 | 0.94 |
| pregnen-diol disulfate* | 0.95 | 0.87 | 0.97 | 0.95 | 0.94 | 0.95 |
| ergothioneine | 0.95 | 0.91 | 0.92 | 0.96 | 0.95 | 0.95 |
| stearamide | 0.15 | 0.27 | 0.17 | 0.032 | 0.067 | 0.21 |
| 9.10-DiHOME | 0.13 | 0.32 | 0 | 0.1 | 0.087 | 0.15 |
| 13-HODE + 9-HODE | 0.12 | 0.44 | 0 | 0.018 | 0 | 0.24 |
| dexpanthenol | 0.11 | 6.50E-15 | 0.16 | 0.27 | 0.026 | 0 |
| xylose | 0.088 | 0.13 | 0 | 0.16 | 0.23 | 0.24 |
| linoleamide (18:2n6) | 0.086 | 0.097 | 0 | 0.14 | 0.11 | 4.90E-16 |
| palmitic amide | 0.081 | 0.11 | 0.018 | 0.11 | 0.035 | 0.0032 |
| oleamide | 0.07 | 0.014 | 0.033 | 0.13 | 0.058 | 4.50E-15 |
| beta-alanine | 0.038 | 0.059 | 0.052 | 0.024 | 0.043 | 0.0075 |
| 3-hydroxy-3-methylglutarate | 0.003 | 0.37 | 0.032 | 0 | 0.16 | 0.12 |

Supplementary Table S3: Metabolites with significant change between visits and with fold-change above 1.5

| **Metabolites** | **FDR** | **Fold-change** |
| --- | --- | --- |
| **Day 14 vs. Day 28** |  |  |
| cytidine | 0.0016 | 1.6 |
| **Day 2 vs. Day 28** |  |  |
| 1,2,3-benzenetriol sulfate (2) | 0.016 | 1.6 |
| TAG42:2-FA12:0 | 0.00085 | 1.6 |
| TAG42:1-FA12:0 | 0.00085 | 1.6 |
| 3-methoxycatechol sulfate (1) | 0.045 | 1.5 |
| TAG40:0-FA12:0 | 0.0035 | 1.5 |
| TAG42:0-FA12:0 | 0.0025 | 1.5 |
| methyl-4-hydroxybenzoate sulfate | 0.005 | 0.66 |
| xylose | 0.04 | 0.64 |
| 5alpha-pregnan-3beta,20alpha-diol disulfate | 0.037 | 0.62 |

Supplementary Figures

Supplementary Figure S1. Characteristics of study participants.


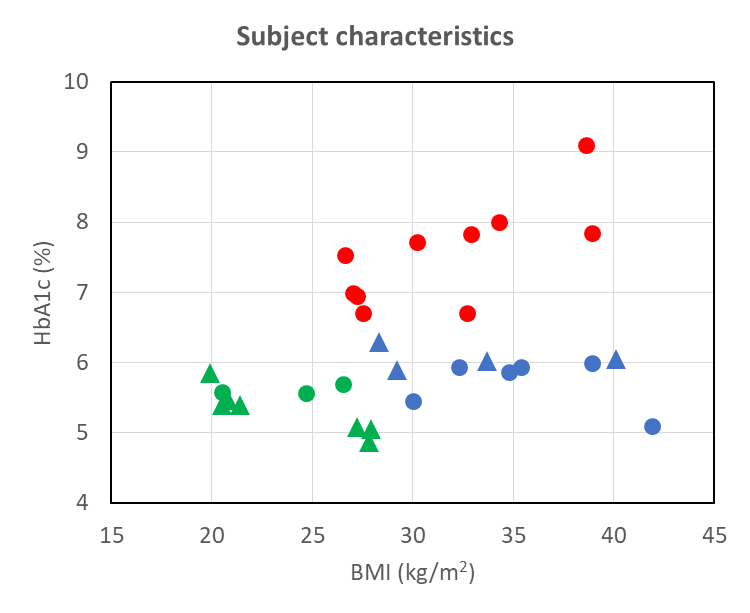


***Green:*** healthy subjects, blue: pre-diabetic individuals, ***red:*** T2D patients. ***Triangles:*** women, ***circles:*** men.

Supplementary Figure S2. OGTT C-peptide profiles at screening


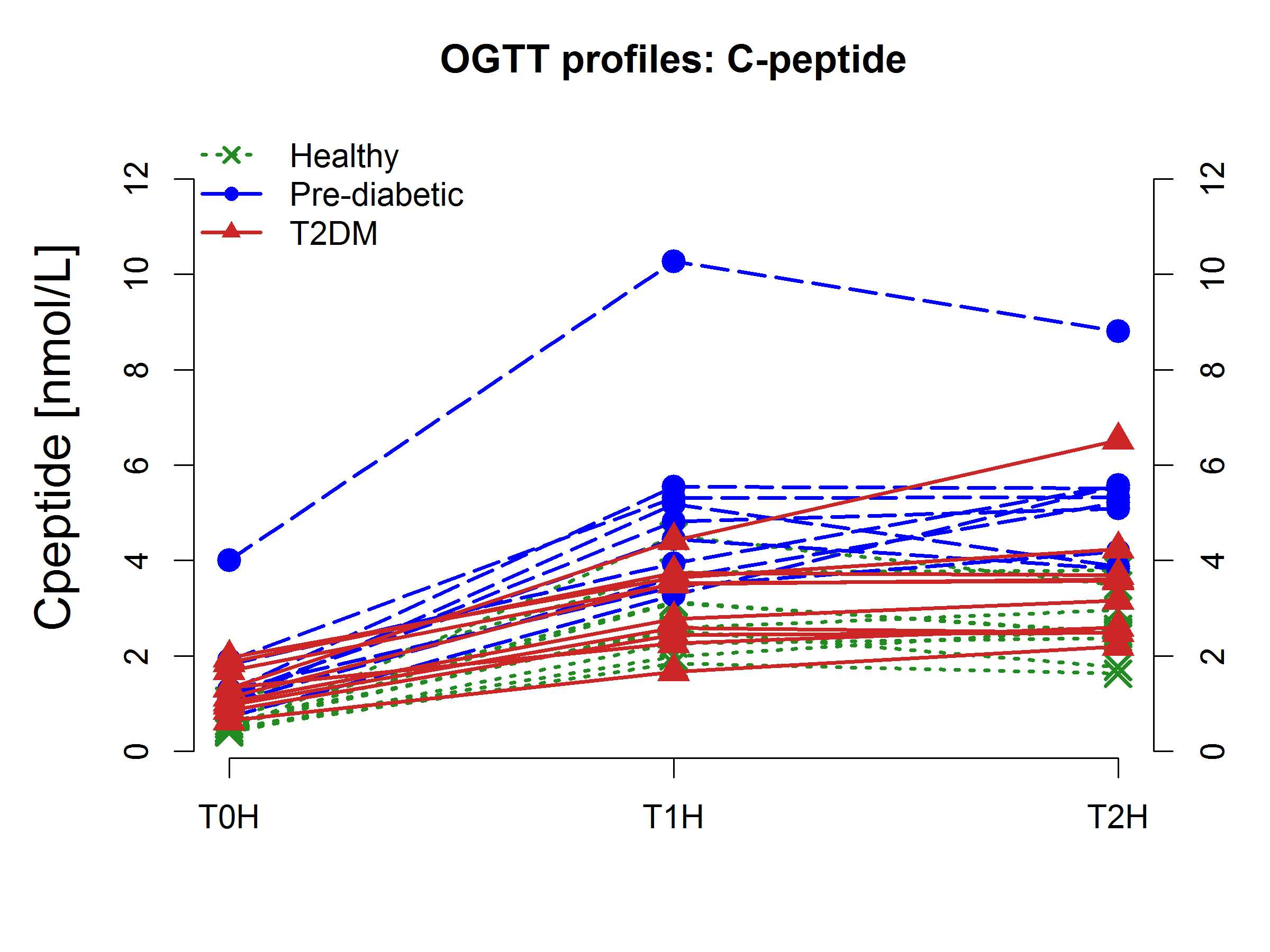


Subject 205

Supplementary Figure S3: Volcano plots for the differences in metabolite levels between study visits. *(a)* Day 2 *vs*. day 14, *(b)* Day 2 *vs*. day 28, *(c)* Day 14 *vs.* day 28.

***(a)***

**
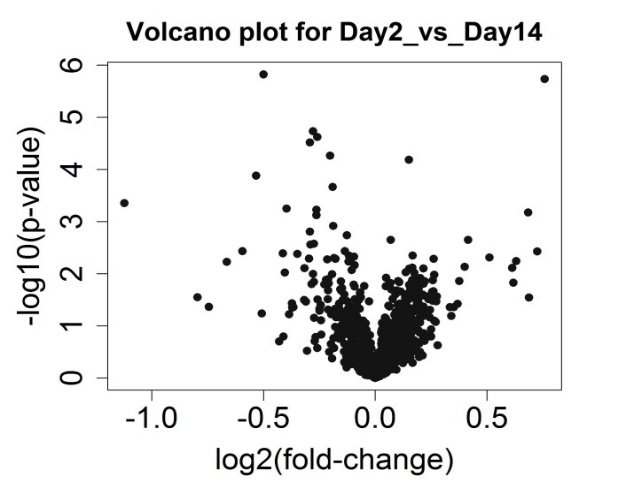
**

***(b)***


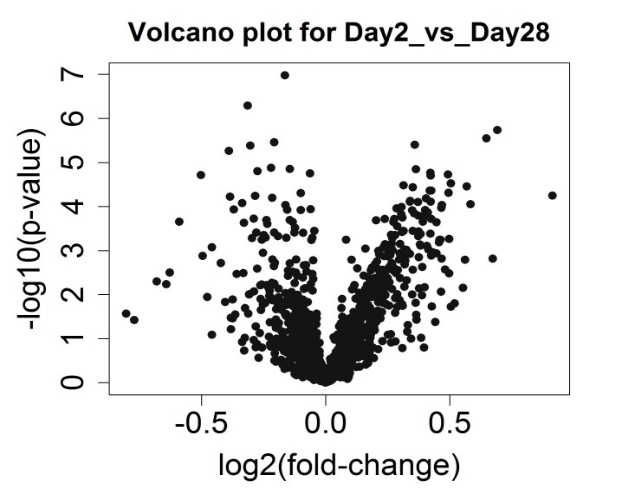


***(c)***


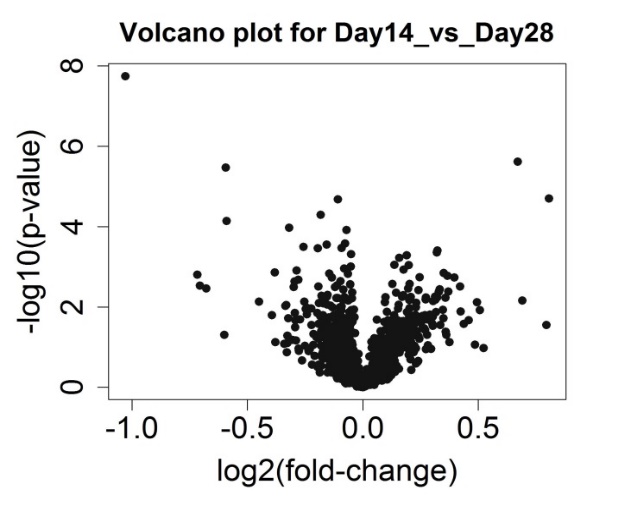


Supplementary Figure S4. 1,5-Anhydroglucitol levels dependent on BMI or HbA1c per health group


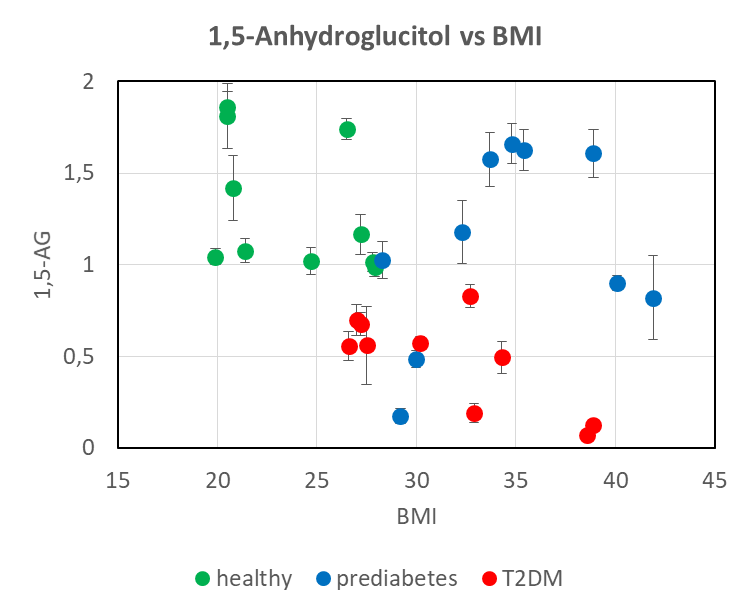

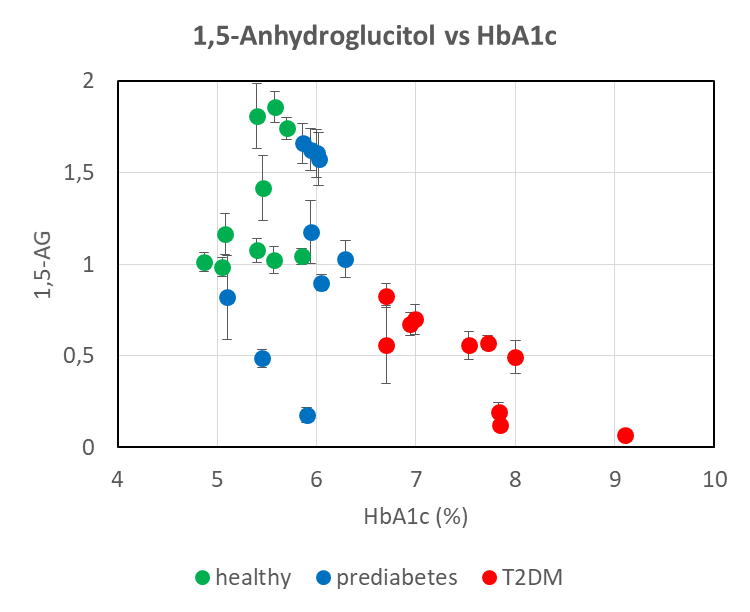


1,5-AG levels are shown as mean ± SD of six measurements on study days 1, 14 and 28, before and after meal intake, respectively, as meal intake had no effect on 1,5-AG levels (cf. figure 9c)

Supplementary Figure S5: Mean profile for acetylcarnitine (C2), glycine and Lysophosphatidylcholine (18:2) by health group


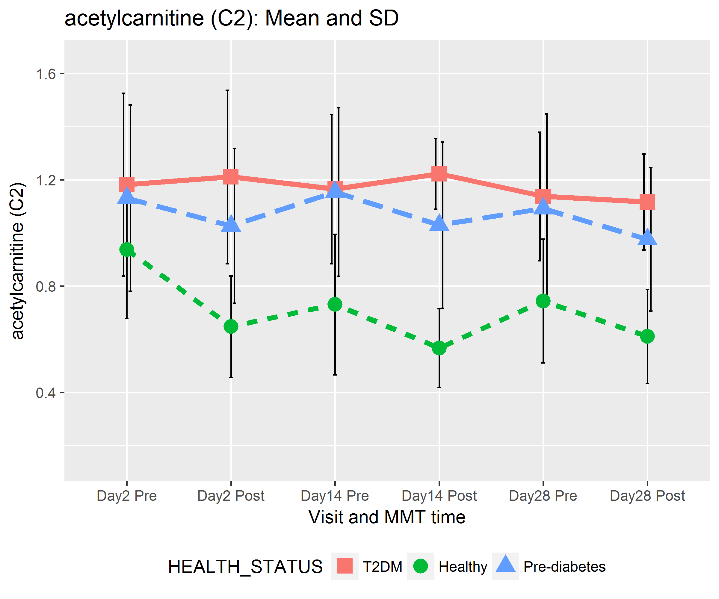

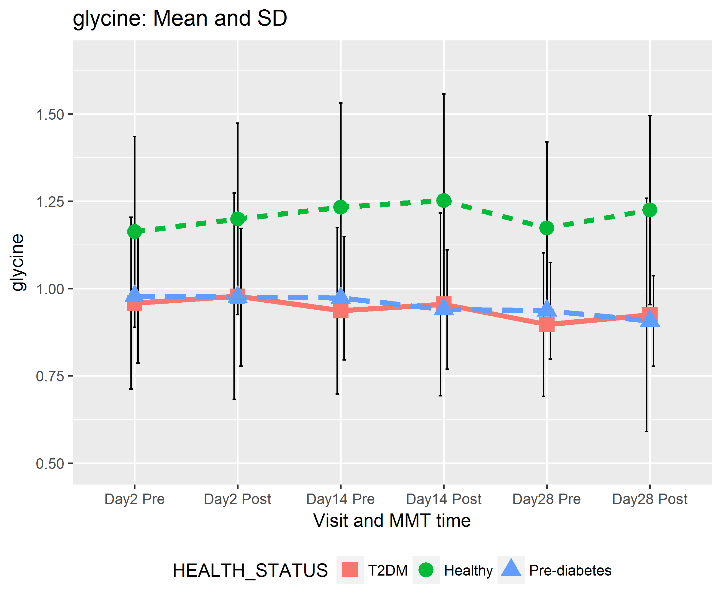


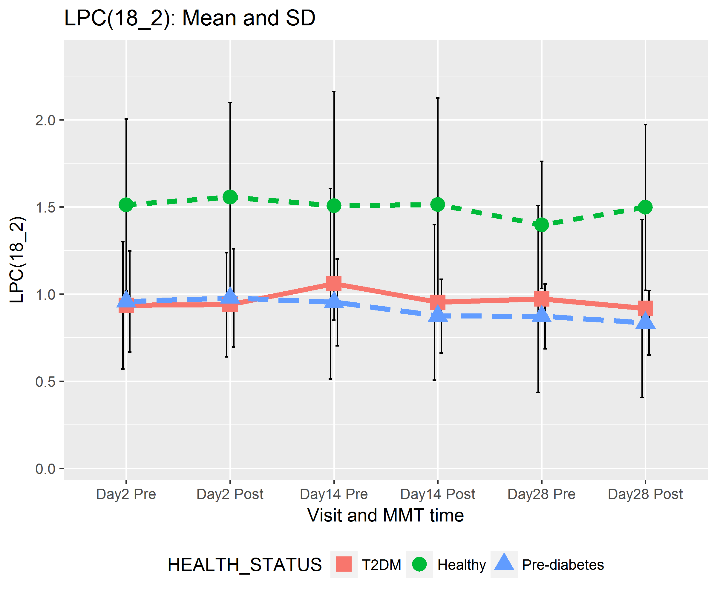


**Supplementary Methods**

**(1) Study Inclusion and Exclusion Criteria**

**1.1 Healthy individuals, inclusion criteria**

***Demography***

IH 01. Male or female individuals, between 18 and 64 years of age, inclusive.

IH 02. Body weight between 50.0 and 100.0 kg, inclusive, if male, and between 40.0 and 90.0 kg, inclusive, if female, body mass index between 18.0 and 28.0 kg/m2, inclusive.

***Health status***

IH 03. Certified as healthy by a clinical assessment (medical history and laboratory assessment).

IH 04. Normal vital signs after 10 minutes resting in supine position:

- 95 mmHg < systolic blood pressure (SBP) ≤ 140 mmHg
- 45 mmHg < diastolic blood pressure (DBP) ≤ 90 mmHg
- 40 bpm < heart rate (HR) ≤ 100 bpm

IH 05. Laboratory parameters within the normal range unless the Investigator considers an abnormality to be clinically irrelevant; however Hb and RBC should not be below the lower laboratory normal limit.

IH 06. Absence of history or presence of clinically relevant cardiovascular, pulmonary, gastrointestinal, hepatic, renal, endocrine, metabolic, hematological, neurological, osteomuscular, articular, psychiatric, systemic, ocular, urologic, gynecologic (if female), or infectious disease, or signs of acute illness. In the discretion of the Investigator, minor dermatologic symptoms may be allowed (e.g., non-acute rosacea, pityriasis versicolor).

IH 07. No intake of any medication (including herbal drugs, over-the-counter (OTC) medication, thyroxin, hormonal contraception and menopausal hormone replacement) during the study and within 14 days prior to screening or within 5 times the elimination half-life or pharmacodynamic half-life of the medication; with the exception of short-term treatment of acute mild clinically disorders (such as intermittent headaches) up to 48 hours before OGTT or MMT visits.

IH 08. Negative result on urine drug screen (amphetamines/methamphetamines, barbiturates, benzodiazepines, cannabinoids, cocaine, opiates).

IH 09. Negative alcohol test.

IH 10. OGTT-2h < 140mg/dL

IH 11. FPG < 100mg/dL

IH 12. HbA1c (glycosylated hemoglobin) < 6.0%

***Regulations***

IH 13. Having given written informed consent prior to undertaking any study-related procedure.

IH 14. Covered by a health insurance system where applicable, and/or in compliance with the recommendations of the national laws in force relating to biomedical research.

IH 15. Not under any administrative or legal supervision.

**1.2 Healthy individuals, exclusion criteria**

***Medical history and clinical status***

EH 01. Frequent headaches and/or migraine, recurrent nausea and/or vomiting (more than twice a month).

EH 02. Blood donation (≥500 mL), within 2 months before inclusion.

EH 03. Presence or history of allergic disease diagnosed and treated by a physician (with the exception of seasonal allergies).

EH 04. History or presence of drug or alcohol abuse (alcohol consumption more than 40 g per day).

EH 05. History of smoking (< 3 months prior to screening). Positive urinary cotinine test.

EH 06. Excessive consumption of beverages containing xanthine bases (more than 6 cups or glasses per day).

EH 07. If female, pregnancy (defined as positive β HCG urine test), breast-feeding.

EH 08. Weight change of ≥5 kg during the last 2 months prior to screening.

EH 09. Any condition possibly affecting gastric emptying or absorption from gastro-intestinal tract (eg, gastric surgery, gastrectomy, bariatric surgery, malabsorption syndromes, abdominal surgery other than appendectomy or hysterectomy).

***Interfering substances***

EH 10. Any vaccination within the last 28 days before screening.

EH 11. Likelihood of requiring treatment during the screening phase and collection phase with drugs not permitted by the clinical study protocol.

***General conditions***

EH 12. Any subject who, in the judgment of the Investigator, is likely to be noncompliant during the study, or unable to cooperate because of a language problem or poor mental development.

EH 13. Any subject in the exclusion period of a previous study according to applicable regulations.

EH 14. Any subject who is the Investigator or any sub-investigator, research assistant, pharmacist, study coordinator, or other staff thereof, directly involved in conducting the study.

***Biological status***

EH 15. Positive result on any of the following tests: hepatitis B surface (HBs Ag) antigen, antihepatitis C virus (anti-HCV) antibodies, anti-human immunodeficiency virus 1 and 2 antibodies (anti-HIV1 Ab and anti HIV2 Ab).

**1.3 Pre-diabetic individuals, inclusion criteria**

***Demography***

IP 01. Male or female individuals, between 18 and 64 years of age, inclusive.

IP 02. Body weight between 50.0 and 160.0 kg, inclusive, if male, and between 40.0 and 150.0 kg, inclusive, if female, body mass index between 18.0 and 40 kg/m2, inclusive.

***Health status***

IP 03. Certified as being pre-diabetic with impaired glucose tolerance with or without comorbidities related to pre-diabetes (e.g., hypertension, cardiovascular, obesity, hyperlipoproteinemia; except if, in the discretion of the Investigator, these conditions hamper study participation) but otherwise healthy by a clinical and laboratory assessment and detailed medical history

IP 04. Normal vital signs after 10 minutes resting in supine position:

- 95 mmHg < systolic blood pressure (SBP) ≤ 160 mmHg
- 45 mmHg < diastolic blood pressure (DBP) ≤ 100 mmHg
- 40 bpm < heart rate (HR) ≤ 100 bpm

IP 05. Laboratory parameters within the normal range unless the Investigator considers an abnormality to be clinically irrelevant or strongly associated with the disease status of prediabetes patients; however Hb and RBC should not be below the lower laboratory normal limit.

IP 06. Absence of any history or presence of clinically relevant cardiovascular, pulmonary, gastrointestinal, hepatic, renal, endocrine, hematological, neurological, osteomuscular, articular, psychiatric, systemic, ocular, gynecologic (if female), urologic, or infectious disease that is not related to the metabolic status of the subject, or signs of acute illness. In the discretion of the Investigator, minor dermatologic symptoms may be allowed (e.g., non-acute rosacea, pityriasis versicolor).

IP 07. No intake of any medication (including herbal drugs, OTC medication, thyroxin, hormonal contraception and menopausal hormone replacement, antihyperlipidemic treatment, antihyperglycemic treatment [e.g. GLP-1 agonists, insulin, glitazones, sulphonyl ureas, etc..]) during the study and within 14 days before OGTT or within 5 times the elimination half-life or pharmacodynamic half-life of the medication, with the exception of:

- antihypertensive treatment (all classes)
- short-term treatment of acute mild clinically disorders (such as intermittent headaches)

up to 48 hours before OGTT or MMT visits.

In case of associated therapy (i.e. antihypertensive treatment only), dose regimen should be the same for each MMT test day (D2, D14 and D28).

IP 08. Negative result on urine drug screen (amphetamines/methamphetamines, barbiturates, benzodiazepines, cannabinoids, cocaine, opiates).

IP 09. Negative alcohol test.

IP 10. HbA1c (glycosylated hemoglobin) ≥ 5.7% and ≤ 6.4%

IP 11. OGTT-2h between 140 and 199 mg/dL

IP 12. FPG between 100 and 125 mg/dL (depending on measured OGTT-1h/-2h glucose values, the sponsor may accept inclusion of individual subjects demonstrating FPG ≥ 90 < 100 mg/dL)

IP 13. Absence of severe dyslipidemia with fasting triglycerides > 450 mg/dL

***Regulations***

IP 14. Having given written informed consent prior to undertaking any study-related procedure.

IP 15. Covered by a health insurance system where applicable, and/or in compliance with the recommendations of the national laws in force relating to biomedical research.

IP 16. Not under any administrative or legal supervision.

**1.4 Pre-diabetic individuals, exclusion criteria**

***Medical history and clinical status***

EP 01. Frequent headaches and/or migraine, recurrent nausea and/or vomiting (more than twice a month).

EP 02. Blood donation (≥500 mL), within 2 months before inclusion.

EP 03. Presence or history of allergic disease diagnosed and treated by a physician (with the exception of seasonal allergies).

EP 04. History or presence of drug or alcohol abuse (alcohol consumption more than 40 g per day).

EP 05. History of smoking (< 3 months prior to screening). Positive urinary cotinine test.

EP 06. Excessive consumption of beverages containing xanthine bases (more than 6 cups or glasses per day).

EP 07. If female, pregnancy (defined as positive β HCG urine test), breast-feeding.

EP 08. Weight change of ≥5 kg during the last 2 months prior to screening.

EP 09. Any condition possibly affecting gastric emptying or absorption from gastro-intestinal tract (eg, gastric surgery, gastrectomy, bariatric surgery, malabsorption syndromes, abdominal surgery other than appendectomy or hysterectomy).

***Interfering substances***

EP 10. Any vaccination within the last 28 days before screening.

EP 11. Likelihood of requiring treatment during the screening phase and collection phase with drugs not permitted by the clinical study protocol.

***General conditions***

EP 12. Any subject who, in the judgment of the Investigator, is likely to be noncompliant during the study, or unable to cooperate because of a language problem or poor mental development.

EP 13. Any subject in the exclusion period of a previous study according to applicable regulations.

EP 14. Any subject who is the Investigator or any sub-investigator, research assistant, pharmacist, study coordinator, or other staff thereof, directly involved in conducting the study.

***Biological status***

EP 15. Positive result on any of the following tests: hepatitis B surface (HBs Ag) antigen, antihepatitis C virus (anti-HCV) antibodies, anti-human immunodeficiency virus 1 and 2 antibodies (anti-HIV1 and anti HIV2 Ab).

**1.5. Type-2 diabetes patients, inclusion criteria**

***Demography***

ID 01. Male or female individuals, between 18 and 64 years of age, inclusive.

ID 02. Body weight between 50.0 and 160.0 kg, inclusive, if male, and between 40.0 and 150.0 kg, inclusive, if female, body mass index between 18.0 and 40 kg/m2, inclusive.

***Health status***

ID 03. Certified as being type 2 diabetes mellitus patients with or without comorbidities related to T2DM (e.g., hypertension, cardiovascular, obesity, hyperlipoproteinemia; except if, in the discretion of the Investigator, these conditions hamper study participation) but otherwise healthy by a clinical and laboratory assessment and detailed medical history.

ID 04. Diagnosis of type 2 diabetes mellitus for at least 1 year at the time of the screening visit.

ID 05. Normal vital signs after 10 minutes resting in supine position:

- 95 mmHg < systolic blood pressure (SBP) ≤ 160 mmHg
- 45 mmHg < diastolic blood pressure (DBP) ≤ 100 mmHg
- 40 bpm < heart rate (HR) ≤ 100 bpm

ID 06. Laboratory parameters within the normal range unless the Investigator considers an abnormality to be clinically irrelevant or strongly associated with the disease status of T2DM patients; however Hb and RBC should not be below the lower laboratory normal limit.

ID 07. Absence of any history or presence of clinically relevant cardiovascular, pulmonary, gastrointestinal, hepatic, renal, endocrine, hematological, neurological, osteomuscular, articular, psychiatric, systemic, ocular, gynecologic (if female), urologic, or infectious disease that is not related to the metabolic status of the subject, or signs of acute illness. In the discretion of the Investigator, minor dermatologic symptoms may be allowed (e.g., non-acute rosacea, pityriasis versicolor).

ID 08. No intake of any medication (including herbal drugs, OTC medication, thyroxin, hormonal contraception and menopausal hormone replacement, antihyperlipidemic treatment, antihyperglycemic treatment [eg, GLP-1 agonists, insulin, glitazones, sulphonyl ureas, etc…]) during the study and within 14 days before OGTT or within 5 times the elimination half-life or pharmacodynamic half-life of the medication, with the exception of:

- metformin (any dose, any regimen - stop from the evening before OGTT/MMT)
- antihypertensive treatment (all classes)
- short-term treatment of acute mild clinically disorders (such as intermittent headaches)

up to 48 hours before OGTT or MMT visits. In case of associated therapy (i.e. metformin or antihypertensive treatment only), dose regimen should be the same for each MMT test day (D2, D14 and D28).

ID 09. Negative result on urine drug screen (amphetamines/methamphetamines, barbiturates, benzodiazepines, cannabinoids, cocaine, opiates).

ID 10. Negative alcohol test

ID 11. HbA1c (glycosylated hemoglobin) ≥ 6.5% and ≤ 9.5%

ID 12. FPG ≥ 126 mg/dL at screening (blood collection before intake of antidiabetic medication in the morning. In patients usually taking an evening metformin dose, the dose at the evening before screening should be omitted).

ID 13. Treatment of T2DM with life style interventions or stable metformin treatment for at least 3 months prior to inclusion

ID 14. Absence of diagnosed diabetic nephropathy, diabetic neuropathy, diabetic retinopathy.

ID 15. Absence of significant renal or hepatic impairment (creatinine >1.5-fold, hepatic enzymes > 3-fold the upper age/gender-related normal laboratory range).

ID 16. Absence of severe hypoglycemia resulting in seizure/unconciousness/coma or hospitalization for diabetic ketoacidosis in the last 3 months before screening.

ID 17. Absence of hyperglycemic episode with coma in the last 3 months before screening.

ID 18. Absence of severe dyslipidemia with fasting triglycerides > 450 mg/dL

***Regulations***

ID 19. Having given written informed consent prior to undertaking any study-related procedure.

ID 20. Covered by a health insurance system where applicable, and/or in compliance with the recommendations of the national laws in force relating to biomedical research.

ID 21. Not under any administrative or legal supervision.

**1.6 Type-2 diabetes patients, exclusion criteria**

***Medical history and clinical status***

ED 01. Frequent headaches and/or migraine, recurrent nausea and/or vomiting (more than twice a month).

ED 02. Blood donation (≥500 mL), within 2 months before inclusion.

ED 03. Presence or history of allergic disease diagnosed and treated by a physician (with the exception of seasonal allergies).

ED 04. History or presence of drug or alcohol abuse (alcohol consumption more than 40 g per day).

ED 05. History of smoking (< 3 months prior to screening). Positive urinary cotinine test.

ED 06. Excessive consumption of beverages containing xanthine bases (more than 6 cups or glasses per day).

ED 07. If female, pregnancy (defined as positive β HCG urine test), breast-feeding.

ED 08. Weight change of ≥5 kg during the last 2 months prior to screening

ED 09. Any condition possibly affecting gastric emptying or absorption from gastro-intestinal tract (eg, gastric surgery, gastrectomy, bariatric surgery, malabsorption syndromes, abdominal surgery other than appendectomy or hysterectomy).

***Interfering substances***

ED 10. Any vaccination within the last 28 days before screening.

ED 11. Likelihood of requiring treatment during the screening phase and collection phase with drugs not permitted by the clinical study protocol.

***General conditions***

ED 12. Any subject who, in the judgment of the Investigator, is likely to be noncompliant during the study, or unable to cooperate because of a language problem or poor mental development.

ED 13. Any subject in the exclusion period of a previous study according to applicable regulations.

ED 14. Any subject who is the Investigator or any sub-investigator, research assistant, pharmacist, study coordinator, or other staff thereof, directly involved in conducting the study.

***Biological status***

ED 15. Positive result on any of the following tests: hepatitis B surface (HBs Ag) antigen, antihepatitis C virus (anti-HCV) antibodies, anti-human immunodeficiency virus 1 and 2 antibodies (anti-HIV1 and anti HIV2 Ab).

**(2) Global Metabolomics Methods**

***Sample Preparation***

Samples were stored at –80°C until processed. Sample preparation was carried out as described previously [1] at Metabolon, Inc. Briefly, recovery standards were added prior to the first step in the extraction process for quality control purposes. To remove protein, dissociate small molecules bound to protein or trapped in the precipitated protein matrix, and to recover chemically diverse metabolites, proteins were precipitated with methanol under vigorous shaking for 2 min (Glen Mills Genogrinder 2000) followed by centrifugation. The resulting extract was divided into five fractions for analysis by ultra-high performance liquid chromatography-tandem mass spectrometry (UPLC-MS/MS): 1) acidic positive ion conditions, chromatographically optimized for more hydrophilic compounds; 2) acidic positive ion conditions, chromatographically optimized for more hydrophobic compounds; 3) basic negative ion optimized conditions using a separate dedicated C18 column; 4) negative ionization following elution from a HILIC column; 5) reserved for backup.

Three types of controls were analyzed in concert with the experimental samples: samples generated from a pool of a small aliquot from each experimental serum sample served as technical replicates throughout the data set; extracted water samples served as process blanks; and a cocktail of standards spiked into every analyzed sample allowed instrument performance monitoring. Instrument variability was determined by calculating the median relative standard deviation (RSD) for the standards that were added to each sample prior to injection into the mass spectrometers (median RSD = 4%; n ≥ 30 standards). Overall process variability was determined by calculating the median RSD for all endogenous metabolites (i.e., non-instrument standards) present in 100% of the pooled human plasma samples (median RSD = 10%; n = 1486 metabolites). Experimental samples and controls were randomized across the platform run.

***Mass Spectrometry Analysis***

Non-targeted MS analysis was performed at Metabolon, Inc. Extracts were subjected to UPLC-MS/MS [1]. The chromatography was standardized and, once the method was validated no further changes were made. As part of Metabolon’s general practice, all columns were purchased from a single manufacturer’s lot at the outset of experiments. All solvents were similarly purchased in bulk from a single manufacturer’s lot in sufficient quantity to complete all related experiments. For each sample, vacuum-dried samples were dissolved in injection solvent containing eight or more injection standards at fixed concentrations, depending on the platform. The internal standards were used both to assure injection and chromatographic consistency. Instruments were tuned and calibrated for mass resolution and mass accuracy daily.

All methods utilized a Waters ACQUITY UPLC and a Thermo Scientific Q-Exactive high resolution/accurate mass spectrometer interfaced with a heated electrospray ionization (HESI-II) source and Orbitrap mass analyzer operated at 35,000 mass resolution. The sample extract was dried then reconstituted in solvents compatible to each of the four methods. Each reconstitution solvent contained a series of standards at fixed concentrations to ensure injection and chromatographic consistency. One aliquot was analyzed using acidic positive ion conditions, chromatographically optimized for more hydrophilic compounds. In this method, the extract was gradient eluted from a C18 column (Waters UPLC BEH C18-2.1x100 mm, 1.7 µm) using water and methanol, containing 0.05% perfluoropentanoic acid (PFPA) and 0.1% formic acid (FA). Another aliquot was also analyzed using acidic positive ion conditions; however, it was chromatographically optimized for more hydrophobic compounds. In this method, the extract was gradient eluted from the same aforementioned C18 column using methanol, acetonitrile, water, 0.05% PFPA and 0.01% FA and was operated at an overall higher organic content. Another aliquot was analyzed using basic negative ion optimized conditions using a separate dedicated C18 column. The basic extracts were gradient eluted from the column using methanol and water, however with 6.5mM Ammonium Bicarbonate at pH 8. The fourth aliquot was analyzed via negative ionization following elution from a HILIC column (Waters UPLC BEH Amide 2.1x150 mm, 1.7 µm) using a gradient consisting of water and acetonitrile with 10mM Ammonium Formate, pH 10.8. The MS analysis alternated between MS and data-dependent MSn scans using dynamic exclusion. The scan range varied slighted between methods but covered 70-1000 m/z.

***Compound Identification, Quantification, and Data Curation***

Metabolites were identified by automated comparison of the ion features in the experimental samples to a reference library of chemical standard entries that included retention time, molecular weight (m/z), preferred adducts, and in-source fragments as well as associated MS spectra and curated by visual inspection for quality control using software developed at Metabolon [2]. Identification of known chemical entities is based on comparison to Metabolon’s spectral library of >4,500 purified chemical standards. Commercially available purified standard compounds have been acquired and registered into LIMS for distribution to the various UPLC-MS/MS platforms for determination of their detectable characteristics. Known metabolites reported in this study conform to confidence Level 1 (the highest confidence level of identification) of the Metabolomics Standards Initiative [3,4], unless otherwise denoted with an asterisk. Additional mass spectral entries have been created for structurally unnamed biochemicals (>2,750 in the Metabolon library), which have been identified by virtue of their recurrent nature (both chromatographic and mass spectral). These compounds have the potential to be identified by future acquisition of a matching purified standard or by classical structural analysis.

Peaks were quantified using area-under-the-curve. Raw area counts for each metabolite in each sample were normalized to correct for variation resulting from instrument inter-day tuning differences by the median value for each run-day, therefore, setting the medians to 1.0 for each run. This preserved variation between samples but allowed metabolites of widely different raw peak areas to be compared on a similar graphical scale.

**References**

1. Evans, A.M., et al. High Resolution Mass Spectrometry Improves Data Quantity and Quality as Compared to Unit Mass Resolution Mass Spectrometry in High-Throughput Profiling Metabolomics. *Metabolomics* **4**, 132 (2014)
2. Dehaven, C.D., Evans, A.M., Dai, H. & Lawton, K.A. Organization of GC/MS and LC/MS metabolomics data into chemical libraries. *J. Cheminform.* **2**, 9 (2010)
3. Sumner, L.W. et al. Proposed minimum reporting standards for chemical analysis Chemical Analysis Working Group (CAWG) Metabolomics Standards Initiative (MSI). *Metabolomics* **3**, 211-221 (2007)
4. Schrimpe-Rutledge, A.C., Codreanu, S.G., Sherrod, S.D. & McLean, J.A. Untargeted metabolomics strategies - challenges and emerging directions. *J. Am. Soc. Mass Spectrom.* **27**, 1897-1905 (2016)
